# Deletion of MIF gene from live attenuated *LdCen*^*−/−*^ parasites enhances protective CD4^+^ T cell immunity

**DOI:** 10.1038/s41598-023-34333-2

**Published:** 2023-05-05

**Authors:** Jacqueline Araújo Fiuza, Sreenivas Gannavaram, Soraya Torres Gaze, Letícia Gambogi de Ornellas, Érica Alessandra Alves, Nevien Ismail, Hira Lal Nakhasi, Rodrigo Correa-Oliveira

**Affiliations:** 1 Cellular and Molecular Immunology Research Group, René Rachou Institute (FIOCRUZ), Belo Horizonte, Brazil; 2 Division of Emerging and Transfusion Transmitted Diseases, Center for Biologics Evaluation and Research, US Food and Drug Administration, Silver Spring, MD USA

**Keywords:** Immunology, Infectious diseases, Vaccines, Parasitology

## Abstract

Vaccination with live attenuated *Leishmania* parasites such as centrin deleted *Leishmania donovani* (*LdCen*^*−/−*^) against visceral leishmaniasis has been reported extensively. The protection induced by *LdCen*^*−/−*^ parasites was mediated by both CD4^+^ and CD8^+^ T cells. While the host immune mediators of protection are known, parasite determinants that affect the CD4^+^ and CD8^+^ T cell populations remain unknown. Parasite encoded inflammatory cytokine MIF has been shown to modulate the T cell differentiation characteristics by altering the inflammation induced apoptosis during contraction phase in experimental infections with *Leishmania* or *Plasmodium*. Neutralization of parasite encoded MIF either by antibodies or gene deletion conferred protection in *Plasmodium* and *Leishmania* studies. We investigated if the immunogenicity and protection induced by *LdCen*^*−/−*^ parasites is affected by deleting MIF genes from this vaccine strain. Our results showed that *LdCen*^*−/−*^*MIF*^*−/−*^ immunized group presented higher percentage of CD4^+^ and CD8^+^ central memory T cells, increased CD8^+^ T cell proliferation after challenge compared to *LdCen*^*−/−*^ immunization. *LdCen*^*−/−*^*MIF*^*−/−*^ immunized group presented elevated production of IFN-γ^+^ and TNF-α^+^ CD4^+^ T cells concomitant with a reduced parasite load in spleen and liver compared to *LdCen*^*−/−*^group following challenge with *L. infantum*. Our results demonstrate the role of parasite induced factors involved in protection and long-term immunity of vaccines against VL.

## Introduction

Visceral leishmaniasis (VL) is considered the second most frequent cause of mortality and the fourth most frequent cause of morbidity within tropical diseases, with 20,000–40,000 deaths per year^1^ and significant economic impact due to an estimated 2 million disability-adjusted life years lost^2^. Strategies to eliminate VL in endemic areas rely on rapid detection and treatment of VL to reduce the number of human reservoirs, and vector control using indoor residual spraying^3^. However, elimination programs in endemic areas have not consistently met the intended milestones, and the need for potent diagnostic, treatment methods and prophylactic vaccines against VL to ensure long term control and prevent reemergence of VL is recognized^3^. Vaccination against VL is considered feasible since long-term protection is acquired following clinical cure of VL in majority of the cases as evidenced by previous studies in VL endemic areas^4–7^. In addition, protection in cutaneus leishmaniasis is observed against infection resulting from the process of leishmanization^5^. A broad range of vaccines including recombinant antigen vaccines, heat-killed parasites, adeno-viral vectored vaccines have been tested against VL^8–13^. Yet no approved vaccine for human VL exists.

We have previously reported on the safety and immunogenicity characteristics of centrin gene deleted live attenuated *Leishmania* parasites (*LdCen*^*−/−*^) as prophylactic vaccines in pre-clinical animal models^14–21^. Gene deleted *Leishmania* parasites such as *LdCen*^*−/−*^ would present a broad repertoire of antigens to generate protective immunity while undergoing limited replication in the immunized host^22^. Since the *LdCen*^*−/−*^ parasite have growth defects in amastigote forms, but not in promastigotes^23,24^, this deletion prevents cell division and long-term persistence in animals (mice and hamsters) or in human macrophages ex vivo^23^. The same non-virulent characteristics of Centrin gene deletion were also observed in other species, like *L. major*, *L. mexicana* and *L. braziliensis*^25–27^. In pre-clinical studies, vaccination with *LdCen*^*−/−*^ proved to be safe, protective and in mice (BALB/c and SCID), hamsters and dogs, after challenge with virulent parasites, in addition to cross-protection in animals challenged with *L. braziliensis, L. infantum* and *L. mexicana*^14–21,28^. Previous studies analyzing the protective immune response following immunization demonstrated the central role of long-lasting Th1-type response^4,9,29–37^. Thus, the induction of a Th1 type response has been considered a pre-requisite in attempts to identify molecules of the parasite and adjuvants that would induce this phenotypic profile in vivo models^9^. Since IFN-γ plays an essential role in the activation of macrophages, allowing the elimination of intracellular pathogens and protecting the host cell against infection^38^, its production is considered one of the main objectives in the immunization process against leishmaniasis. Accordingly, the protective immunity induced by *LdCen*^*−/−*^ parasites has been shown to be mediated by Th1 dominant multifunctional CD4^+^ and CD8^+^ T cell populations that orchestrate the assembling of granulomas in the spleens followed by the parasiticidal activities mediated by nitric oxide^14^. Recent studies also showed that in a centrin deletion mutant of *L. major*, skin resident TRM (Tissue Resident Memory) cells also play an important role in protection^39^. Towards understanding the immune mechanisms that direct the development of Th1-type response following *LdCen*^*−/−*^ immunization, studies investigated early interactions between *LdCen*^*−/−*^ parasites and the innate immune cells. These studies revealed reprogramming of M1/M2 macrophages, reconfiguration of the membrane architecture to enhance cholesterol driven fluidity, variable co-stimulatory and co-inhibitory ligands, and chemokine/cytokine signatures and the corresponding regulatory microRNAs enabling the development of Th1 type immune response following *LdCen*^*−/−*^ immunization compared to infection with virulent *LdWT* parasites^14,40–42^. Yet, while the immunological characteristics of the innate and adaptive responses showed significant differences between *LdCen*^*−/−*^ and *LdWT* infections, the parasitic factors that drive these changes remain to be understood.

As the mediators of protection, the characteristics of CD4^+^ and CD8^+^ T cell memory subpopulations and their role in prophylaxis or treatment^43–51^ has been studied thoroughly. A study evaluating different types of T cells in visceral leishmaniasis, including memory T cells without identifying subtypes, demonstrated a lower number of memory T cells in patients with VL compared with treated or asymptomatic patients^52^. Different combinations of effector memory (T_EM_)/central memory (T_CM_) T cells have been shown to be important in inducing protection against secondary infections by *Leishmania*^53–57^. Thus, successful vaccination must be based on a low dose of antigen, to allow slow replication of effector cells and favor differentiation of memory T cell populations^57,58^.

Studies in *Leishmania* and *Plasmodium* identified a parasite-encoded ortholog of a cytokine macrophage migration inhibition factor (MIF) that has been shown to affect the macrophage apoptosis, activation signals, CD4 T cell apoptosis, and CD4 effector T cell responses^59–62^. Evidence indicates that *Leishmania* encoded MIF cytokine may drive an inflammatory environment that is detrimental to the host response^63^. Deletion of MIF in *Leishmania major* parasites showed that antigen presenting, T cell priming and the IFN-γ, IL-7R production by CD4 T cells were significantly affected due to the loss of MIF genes^63^. Studies in *Plasmodium berghei* showed that *Plasmodium* encoded MIF enhanced inflammatory cytokine production and induced antigen experienced CD4^+^ T cells to develop into short-lived effector cells rather than memory precursor cells. The short-lived effector CD4 T cells were more readily eliminated by Bcl-2-associated apoptosis, resulting in decreased CD4 T-cell recall responses against challenge infections^61^. Thus, to investigate the role of *Leishmania* encoded MIF in generating effector and memory T cell populations following *LdCen*^*−/−*^ immunization, we created additional deletion of MIF genes (MIF1 and MIF2, tandemly arranged MIF genes) in the vaccine strain and analyzed the long-term immune response. *Leishmania* MIF has been shown to interact with its receptor CD74 and exhibit an anti-apoptotic activity that may facilitate the intracellular persistence of the parasite in macrophages^59^. Thus, the MIF deletion would likely increase the long-term immune response that is essential for a successful VL vaccine.

We have evaluated the profile of memory T cells after immunization with the *Leishmania donovani* double-knock out parasites (*LdCen*^*−/−*^*MIF*^*−/−*^) and single attenuated (*LdCen*^*−/−*^*)* parasites. Immunized mice with both types of parasites demonstrated a strong immune response, capable of inducing T_CM_ populations following immunization. The changes in the profile of T_EM_ populations after challenge were also observed, resulting in significant cross-protection against infection with virulent *L*. *infantum* parasites.

## Results

### LdMIF proteins induce inflammatory cytokines

**Figure 1:**
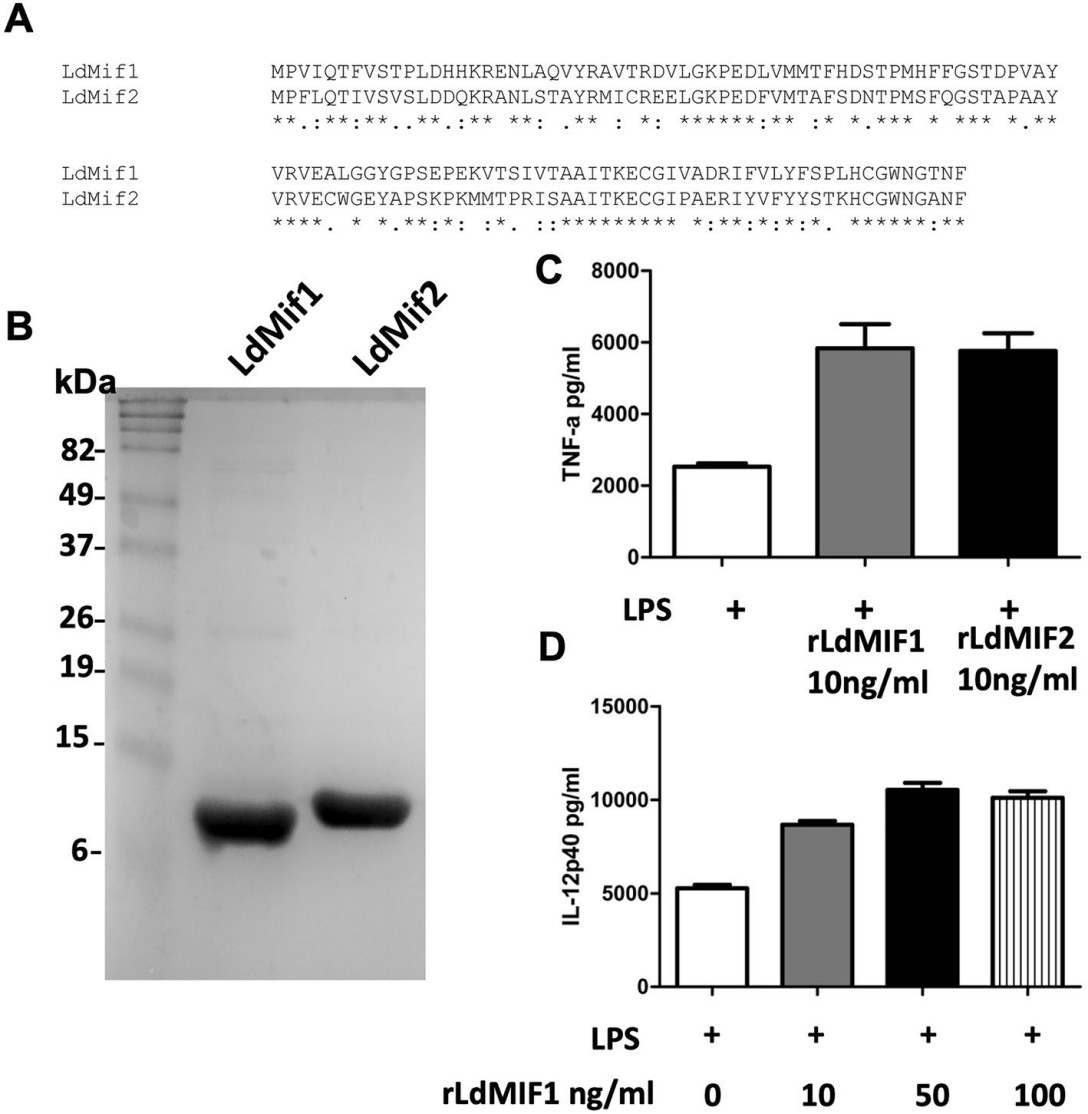
Cytokines production after stimulation with LdMIF1 and LdMIF2 proteins. (**A**) *L. donovani* sequences of MIF genes. (**B**) Coomassie stained gel showing LdMIF1 and LdMIF2 proteins purified. (**C**) TNF-α and (**D**) IL-12p40 production after stimulation of C57BL/6 murine BMDCs with purified recombinant LdMIF1 and LdMIF2 proteins, compared to positive control (LPS). The experiment was performed four times (n = 3/replication), and cells were pooled and the error bars represent the standard deviation of each group.

*Leishmania donovani* genome contains two tandemly arranged copies of MIF genes that share significant homology (Fig. 1A). The two ORFs corresponding to MIF genes were PCR amplified and used to produce recombinant proteins. Coomassie stained gel showed LdMIF1 and LdMIF2 proteins were purified to a high degree (Fig. 1B). To test whether LdMIF proteins induce inflammatory cytokines similar to other parasitic protozoa, purified recombinant LdMIF1 and LdMIF2 proteins were incubated with murine BMDCs. Culture supernatants showed significant induction of TNF-α in presence of either LdMIF1 or LdMIF2 proteins relative to untreated control (Fig. 1C). Similarly, BMDC cultures incubated with rLdMIF1 and rLdMIF2 (not shown) also showed an induction of IL-12 in a dose dependent manner (Fig. 1D). Thus, we observed that LdMIF1 and LdMIF2 proteins were able to induce a proinflammatory response.

### Deletion of MIF genes does not affect the amastigote proliferation

**Figure 2:**
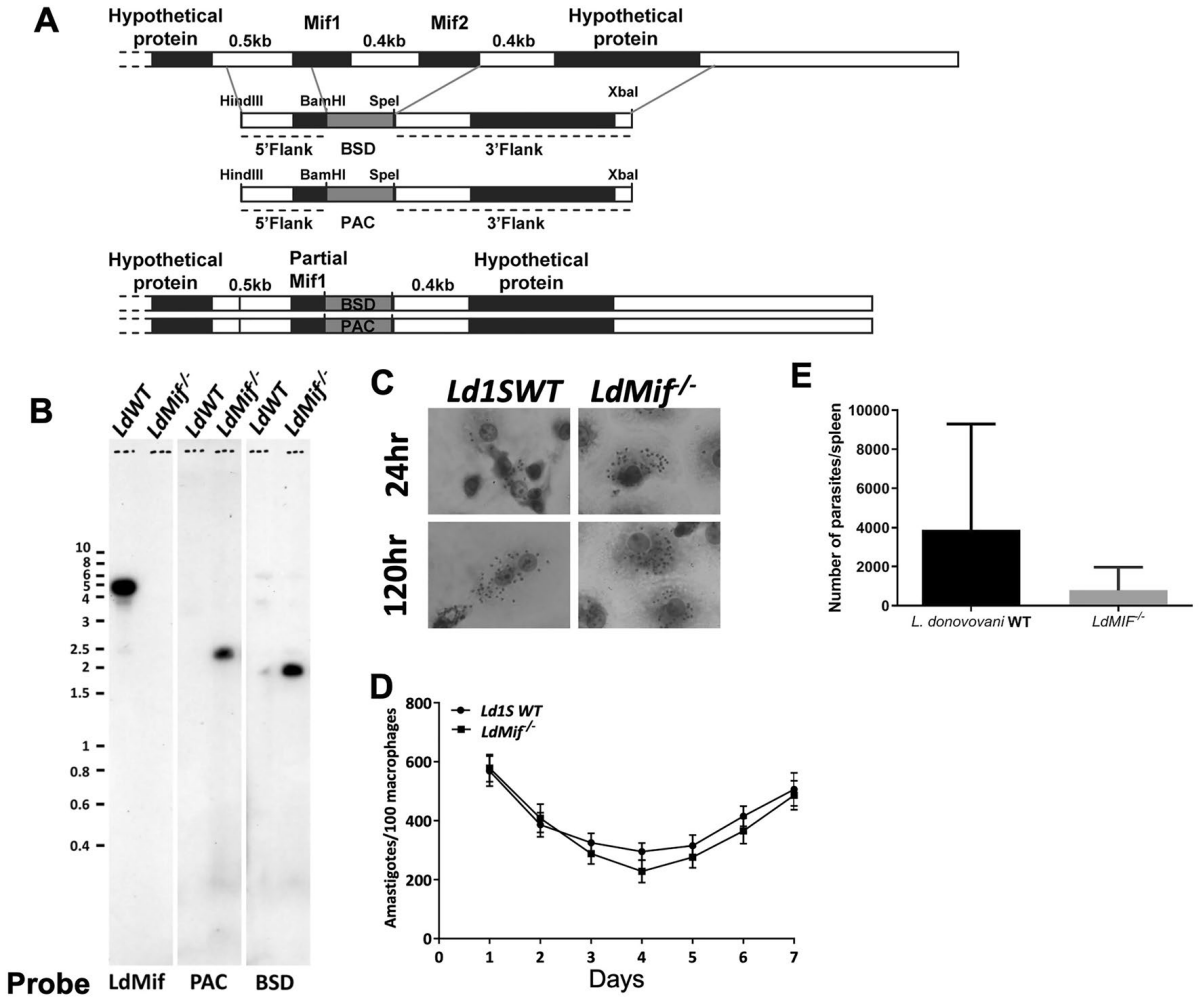
Deletion of MIF genes. (**A**)The two tandemly arranged copies of LdMIF genes and the genomic context showing the gene replacement construct containing either Blasticidin (BSD) or Puromycin (PAC) targeted deletion of both copies of MIF genes including the intergenic region. (**B**) Southern blot of the transfected (autoradiograph). (**C**) Microscopy of the murine macrophages infected with *LdWT* and *LdMIF*^*−/−*^ parasites to evaluate the effect of fitness and growth as amastigotes. (**D**) Number of parasites/100 macrophages infected with *LdMIF*^*−/−*^ parasites, compared to *LdWT* parasites during 7 consecutive days. (**E**) Number of parasites per spleen of BALB/c mice 4 weeks after infection with *LdWT* and *LdMIF*^*−/−*^ parasites. The experiment was performed four times (n = 3/replication), and cells were pooled. The error bars represent the standard deviation of each group.

To test the effect of the LdMIF induced inflammatory response in immunogenicity, we prepared MIF deletion mutants. The two tandemly arranged copies of LdMIF genes and the genomic context are shown (Fig. 2A). The gene replacement construct contained either Blasticidin or Puromycin targeted deletion of both copies of MIF genes including the intergenic region. The MIF genes were deleted in a sequential transfection with Blasticidin and puromycin constructs and selection with respective antibiotics. Southern hybridization with the indicated probes showed the deletion of both MIF genes from *LdWT* genome (*LdMIF*^*−/−*^ lane, Fig. 2B).

To test whether deletion of MIF genes affects the parasite fitness and growth as amastigotes, murine macrophages were infected with *LdWT*, *LdMIF*^*−/−*^ parasites (Fig. 2C). Results showed that deletion of MIF genes does not affect the growth of amastigotes as their growth paralleled that of *LdWT* parasites (Fig. 2D). To test if MIF deletion affects the parasite replication in vivo, mice were infected with *LdMIF*^*−/−*^ parasites and compared to mice infected with *L. donovani* WT. After 4 weeks, splenic burden was measured. Results showed that *LdMIF*^*−/−*^ infected mice presented parasites in spleen (Fig. 2E). Deletion of *MIF* in *L. donovani* parasites does not affect replication. However, we observed less parasites (not statistically significant) in spleen of *LdMIF*^*−/−*^ infected mice. It could indicate that the mutant parasites grow slower than wild type strain but further studies are necessary.

### MIF deletion results in reduced CD4^+^ T cell apoptosis

**Figure 3:**
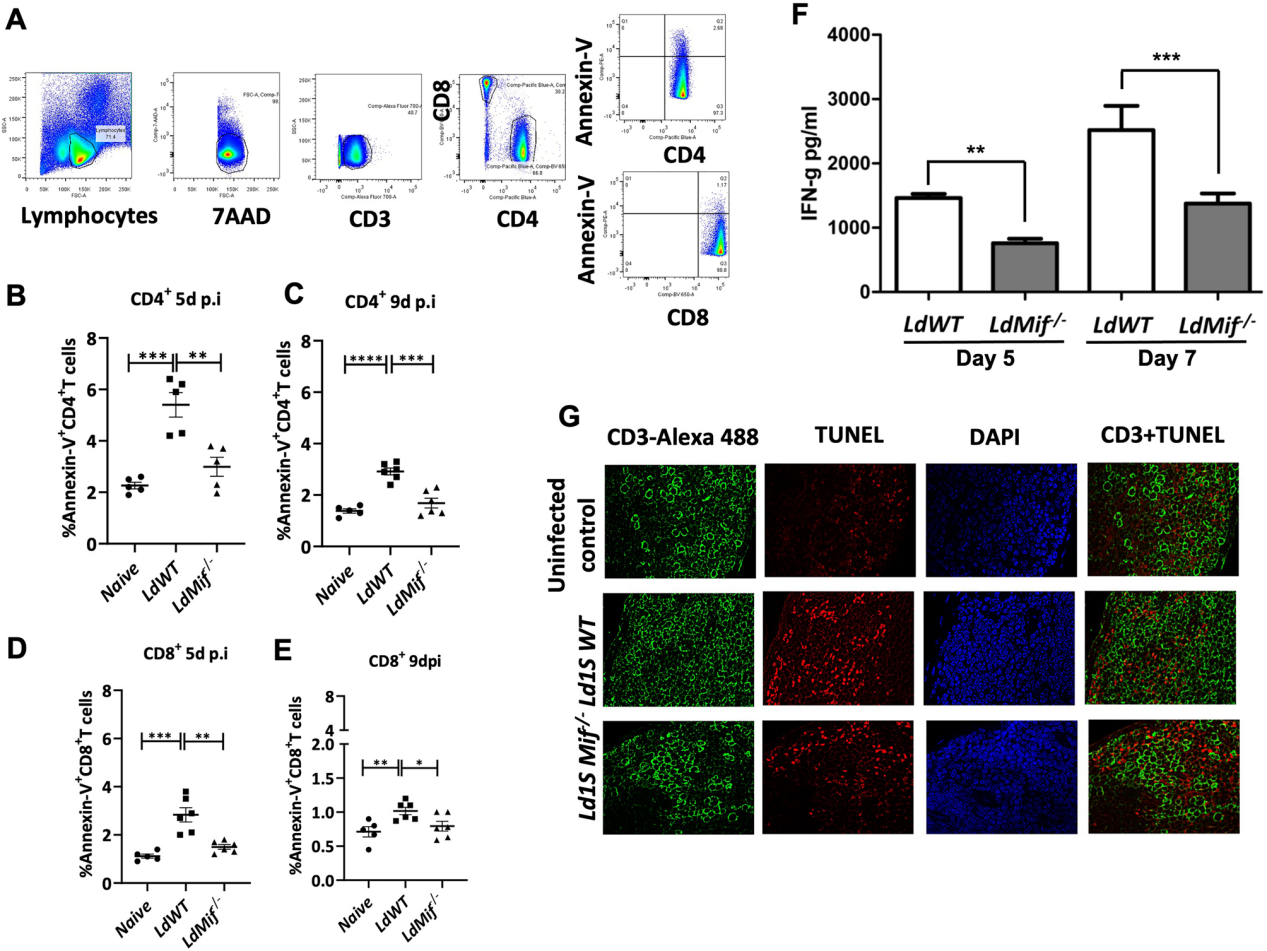
Apoptosis profile of CD4^+^ T cells in mice infected with *LdMIF*^*−/−*^ parasites. (**A**) Gate strategy for flow cytometry. Apoptosis evaluation using Annexin-V^+^ as a marker in (**B**) and (**C**) CD4^+^ and (**D**) and (**E**) CD8^+^ T cells, at 5 (**B** and **D**) and 9 (**C** and **E**) days post infection. Significant differences are indicated on the graphs (*p < 0.05; **p < 0.01; ***p < 0.001 and ****p < 0.0001). (**F**) IFN-γ production from splenocytes of *LdMIF*^*−/−*^ and *LdWT* infected mice at days 5 and 7 post injection. 0.25 µg/ml LPS was used in the stimulation. (**G**) Expression of the anti-apoptotic Bcl2 protein in splenocytes from *LdMIF*^*−/−*^ and *LdWT* infected BALB/c mice. (**H**) Immunohistochemistry of the spleens from *LdWT* and *LdMIF*^*−/−*^ at 9 days post infection. The cells were labelled with TUNEL^+^, DAPI and CD3-Alexa 488. The experiment was performed four times (n = 3/replication), and cells were pooled. The error bars represent the standard deviation of each group.

To test whether MIF deficiency results in reduced inflammation and thus diminished T cell apoptosis in vivo, we infected Balb/C mice with *LdWT*, and *LdMIF*^*−/−*^ parasites. T cell apoptosis in the spleens of the infected mice was monitored by flow cytometry as shown (Fig. 3A). Results showed that the CD4^+^ T cell apoptosis (Annexin-V^+^) was significantly reduced on days 5 and 9 of the *LdMIF*^*−/−*^ infection compared to *LdWT* infection (Fig. 3B,C, respectively) corresponding to the expansion phase of the T cell responses post-infection. No significant difference was observed in the Annexin^+^ CD4 T cell population on day14, that corresponds to the post-contraction phase (data not shown). Similar differences in CD8^+^ T cell populations were also evident to a lesser degree in *LdMIF*^*−/−*^ infection (Fig. 3D,E). Correspondingly, IFN-γ production from the splenocytes of *LdMIF*^*−/−*^ infected mice showed a significant reduction compared to *LdWT* infection after 5 (p = 0.01) and 7 (p = 0.001) days (Fig. 3F). Immunohistochemistry of the spleens from *LdWT*, and *LdMIF*^*−/−*^ 9 days post infection showed the presence of TUNEL^+^ cells in *LdWT* but much less in *LdMIF*^*−/−*^ infection (Fig. 3G) indicating that deletion of MIF promotes the survival of T cells in the infected spleens consistent with our flow cytometric analysis.

### Deletion of MIF in *LdCen*^−/−^ background

**Figure 4:**
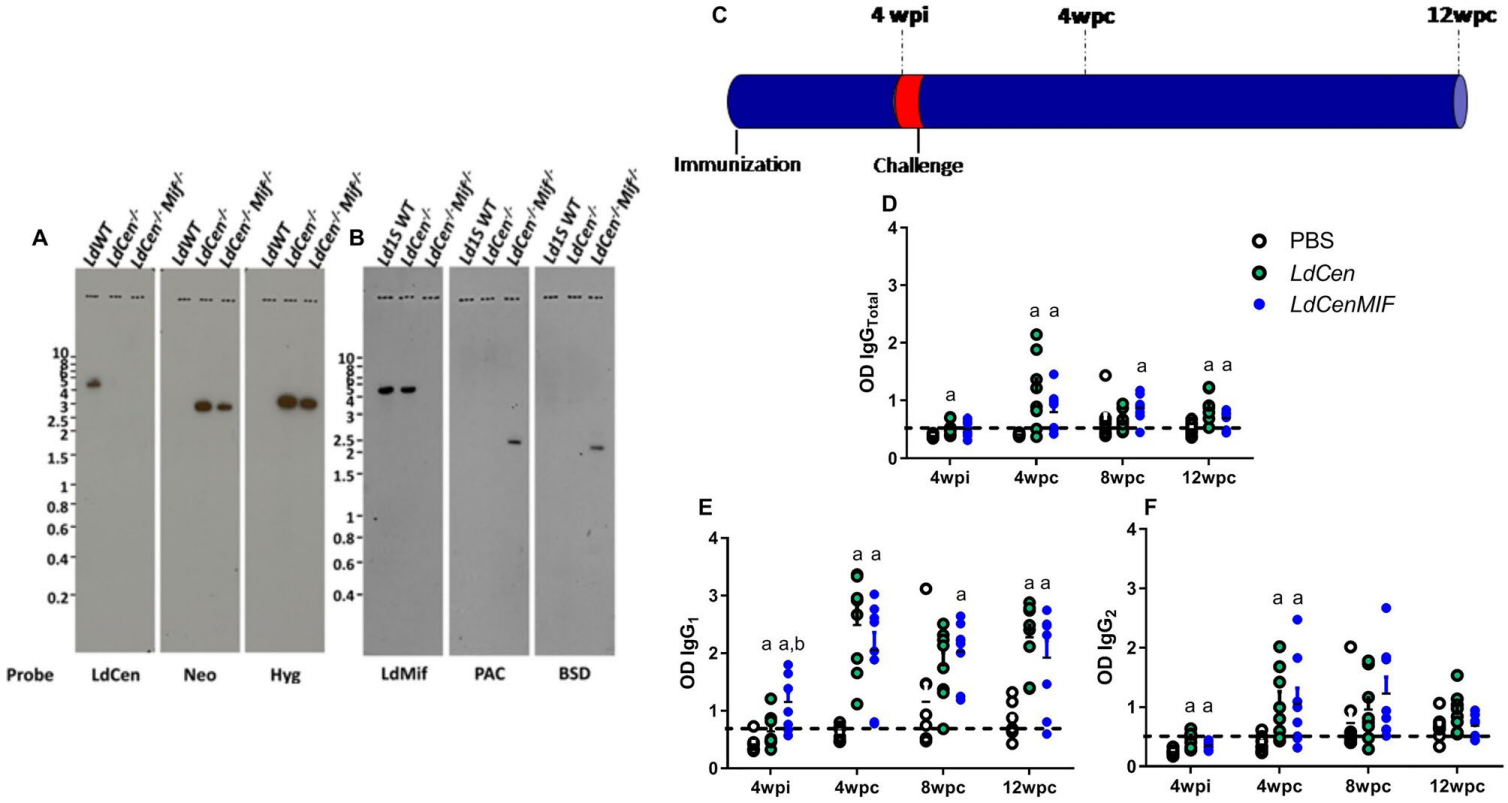
MIF deletion in the *LdCen*^*−/−*^ mutant background and specific antibody production. (**A**) and (**B**) Southern blot of MIF deletion in the *LdCen*^*−/−*^ mutant background (autoradiograph). (**C**) Immunization scheme. (**D**–**F**) ELISAs using plates coated with SLA from *L*. *infantum* were performed to detect production of total IgG (**D**), IgG_1_ (**E**) and IgG_2_ (**F**). The antibody OD values are shown on the *y*-axis, and the error bars indicate the standard deviation. Dotted lines represent the cut-off value. Statistical differences (*p* < 0.05) are indicated in letters (a: PBS; b: *LdCen*^*−/−*^). The animal experiments were performed in three replicates and the error bars represent the standard deviation of each group (n = 8 mice/group). The BALB/c mice were evaluated individually.

Since MIF deletion in *L. donovani* showed enhanced survival of CD4^+^ T cells in spleens as was shown in studies with virulent *L. major* parasites^60,63^, we performed MIF deletion in the *LdCen*^*−/−*^ mutant background to test if the quality of T cell responses can be tweaked further towards inducing strong vaccine induced protection. Using gene targeting strategy described above, we deleted MIF genes from *LdCen*^*−/−*^ mutant. Southern hybridization with the indicated probes showed the deletion of both MIF genes from *LdCen*^*−/−*^ genome (*LdCen*^*−/−*^*MIF*^*−/−*^ lane, Fig. 4A,B). Hybridization with Neo and Hyg probes showed that *LdCen*^*−/−*^ genotype remained unperturbed due to MIF deletion (Fig. 4B).

To test the immunogenicity of *LdCen*^*−/−*^*MIF*^*−/−*^ double deletion mutants, we immunized mice with *LdCen*^*−/−*^ or *LdCen*^*−/−*^*MIF*^*−/−*^ mutants (Fig. 4C). Anti-*Leishmania* vaccines induce humoral immune response that are often used as a surrogate of adaptive immunity which is the main driver of protection. The ability of the attenuated parasites to induce IgG_Total_, IgG_1_ and IgG_2_ antibodies against soluble antigen of *Leishmania infantum*, after immunization or challenge, was investigated (Fig. 4D–F). The profile of antibodies was measured 4 weeks post-immunization (4wpi), as well as 4, 8 and 12 weeks after the challenge (wpc). Immunization with the double-attenuated strain *LdCen*^*−/−*^*MIF*^*−/−*^ increased the secretion of IgG_Total_ at 8wpc (p < 0.01) (Fig. 4D) and IgG_1_ at 4wpi and 8wpc (p < 0.01) (Fig. 4E). In addition, both *LdCen*^*−/−*^ and *LdCen*^*−/−*^*MIF*^*−/−*^ attenuated parasites were able to induce higher levels of IgG_Total_ (4wpc and 12wpc) (p < 0.01) (Fig. 4D), IgG_1_ (4wpi, 4 and 12wpc) (p < 0.01, p < 0.001 and p < 0.01, respectively) (Fig. 4E), and IgG_2_ (4wpi and 4wpc) (Fig. 4F) compared to non-immunized group. The results suggest an induction of cross-reactive antibodies since the animals were immunized with *L. donovani* attenuated parasites and challenged with a *L. infantum* strain.

### Effect of MIF deletion on T cell proliferation in *LdCen*^*−/−*^*MIF*^*−/−*^ immunized mice

**Figure 5:**
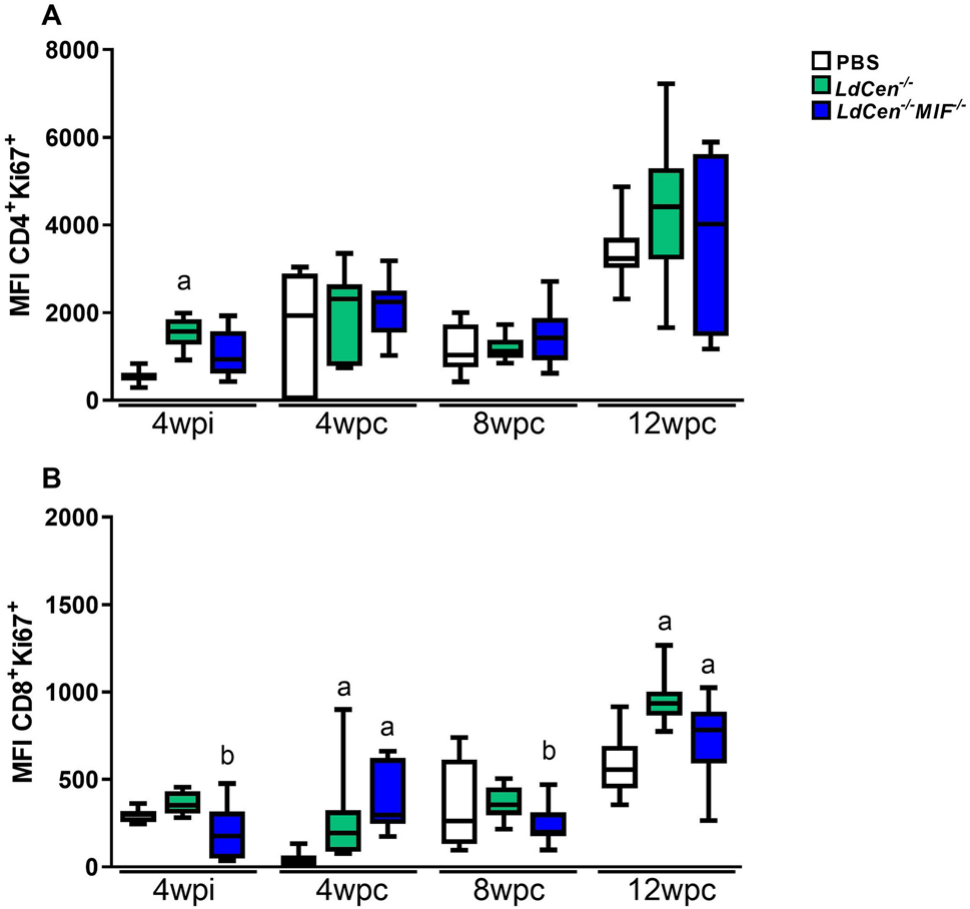
T Cell proliferative responses in animals immunized with attenuated parasites. Proliferation of T CD4^+^ (**A**) and T CD8^+^ (**B**) cells after pulsing with *L. infantum* SLA for 72 h. Proliferation responses were expressed in terms of Medium Intensity of Fluorescence (MIF) of Ki67 marker. Significant differences are indicated on the graphs (a: PBS; b: *LdCen*^*−/−*^). The experiments were performed in three replicates and the error bars represent the standard deviation of each group (n = 8 mice/group). The BALB/c mice evaluated individually.

In order to assess if vaccination with single dose of *LdCen*^*−/−*^ or *LdCen*^*−/−*^*MIF*^*−/−*^ parasites (before and after challenge with *L. infantum* WT) had an effect on T cell function, we first evaluated the capacity of splenocytes T cells to proliferate upon specific SLA stimulation. The profile of proliferation was evaluated at 4wpi, 4wpc and 12wpc. For this, we used flow cytometry to measure the mean intensity fluorescence of Ki67 in CD4^+^ and CD8^+^ T cells (Fig. 5). We observed a significant increase of proliferation of CD4^+^ and CD8^+^ T cells on groups immunized with *LdCen*^*−/−*^ at 4wpi (p < 0.01) (Fig. 5A) and of CD8^+^ T cells on group immunized with *LdCen*^*−/−*^*MIF*^*−/−*^ at 4wpi (p = 0.024) (Fig. 5B). For CD4^+^ T cell, in vitro stimulation with SLA did not increase proliferation after challenge. In fact, after 4wpi there was a decrease in proliferation of CD4^+^ T cells under SLA stimulation (p < 0.01) (Fig. 5A). In CD8^+^ T cell subpopulation, double-attenuated strain *LdCen*^*−/−*^*MIF*^*−/−*^ decreased proliferation at 4wpi (Fig. 5A), but after the challenge with *L. infantum* this group of cells was able to proliferate in vitro under SLA stimulation (p < 0.01) (Fig. 5B).

### Intracellular cytokines expression by T cells in *LdCen*^*−/−*^*MIF*^*−/−*^ immunized mice

**Figure 6:**
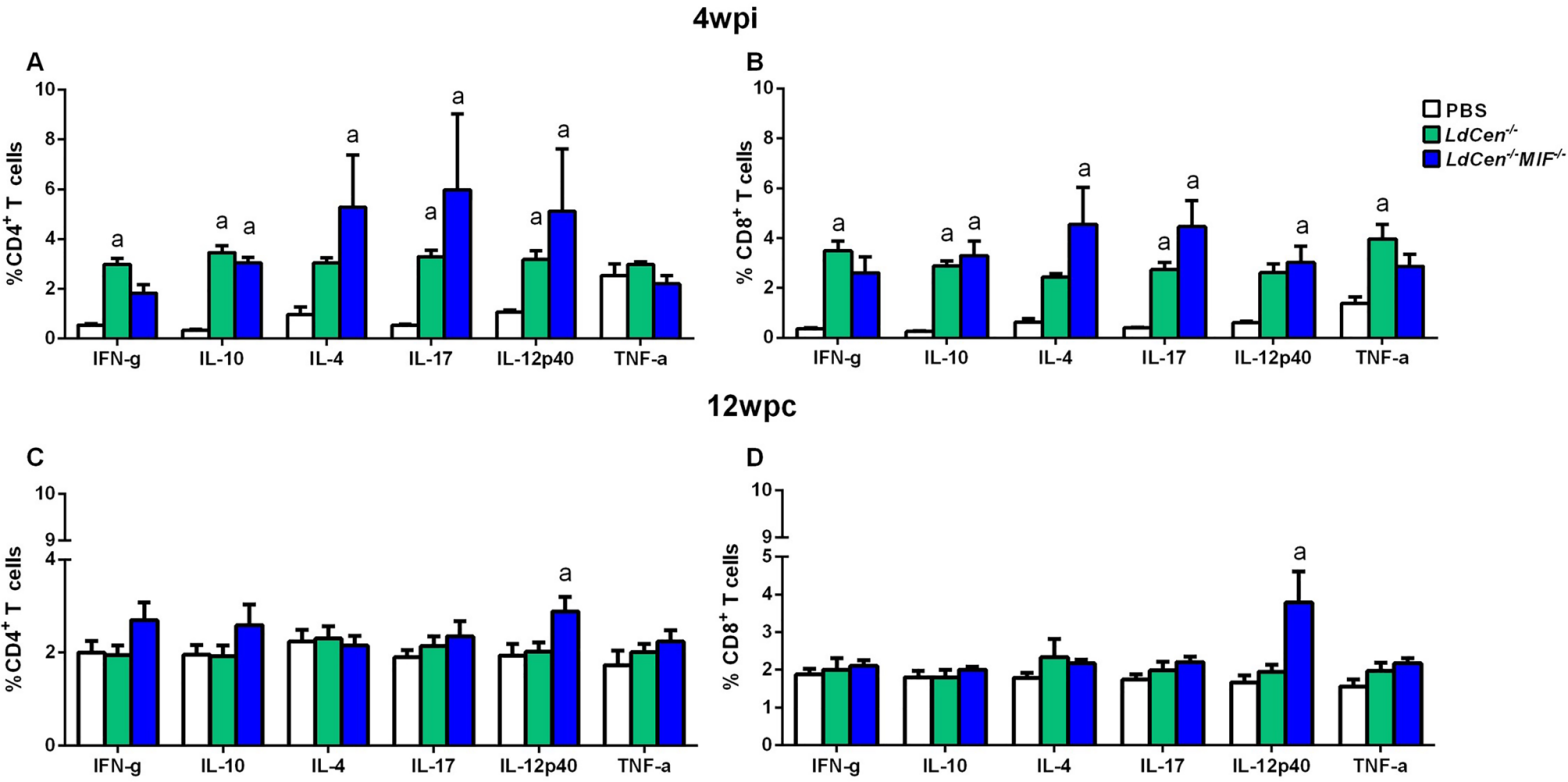
Cytokines production by T cells. Percentage of IFN-γ, IL-12/IL-23p40, TNF-α, IL-17A, IL-10 and IL-4 production was assessed in the stimulated CD4^+^ (**A** and **C**) and CD8^+^ (**B** and **D**) T cells, at 4 weeks post immunization and 12 weeks post challenge. Results from cultures are expressed as ratio (percentage of cultures stimulated with SLA *L. infantum*/Percentage of unstimulated cultures). Significant differences are indicated on the graphs (a: PBS). The experiments were performed in three replicates and the error bars represent the standard deviation of each group (n = 8 mice/group). The BALB/c mice were evaluated individually.

Having observed that immunization with *LdCen*^*−/−*^ or *LdCen*^*−/−*^*MIF*^*−/−*^ attenuated parasites leads to proliferation of mostly CD8^+^ T cells after SLA stimulation, we evaluated the profile of cytokines (IFN-γ, IL-10, IL-4, IL-17, IL-12p40 and TNF-α) expression by CD4^+^ and CD8^+^ T cells. The mice immunized with *LdCen*^*−/−*^ or *LdCen*^*−/−*^*MIF*^*−/−*^ were compared to the PBS group, 4 weeks post immunization (4wpi) and 12 weeks post challenge (12wpc). The analyses strategy can be observed in supplementary Fig. 2. As shown in Fig. 6A, there was an increase of the percentage of IL-10, IL-4, IL-17 and IL-12/IL-23p40 by both CD4^+^ and CD8^+^ T cells from *LdCen*^*−/−*^*MIF*^*−/−*^ immunized animals at 4wpi, when compared to PBS group (p < 0.01) (Fig. 6A,B). However, there was increase in TNF-α only in CD8^+^ T cells at 4 wpi in *LdCen*^*−/−*^ group (Fig. 6B). At 12wpc, we observed only high expression of IL-12p40 by both CD4^+^ and CD8^+^ T cells in *LdCen*^*−/−*^*MIF*^*−/−*^ group, compared to PBS group (p < 0.01) (Fig. 6C,D, respectively). We have observed a mixed profile of cytokine expression by T cells after both immunization and challenge.

### Effect of MIF deletion on memory T cell populations in *LdCen*^*−/−*^*MIF*^*−/−*^ immunized mice

**Figure 7:**
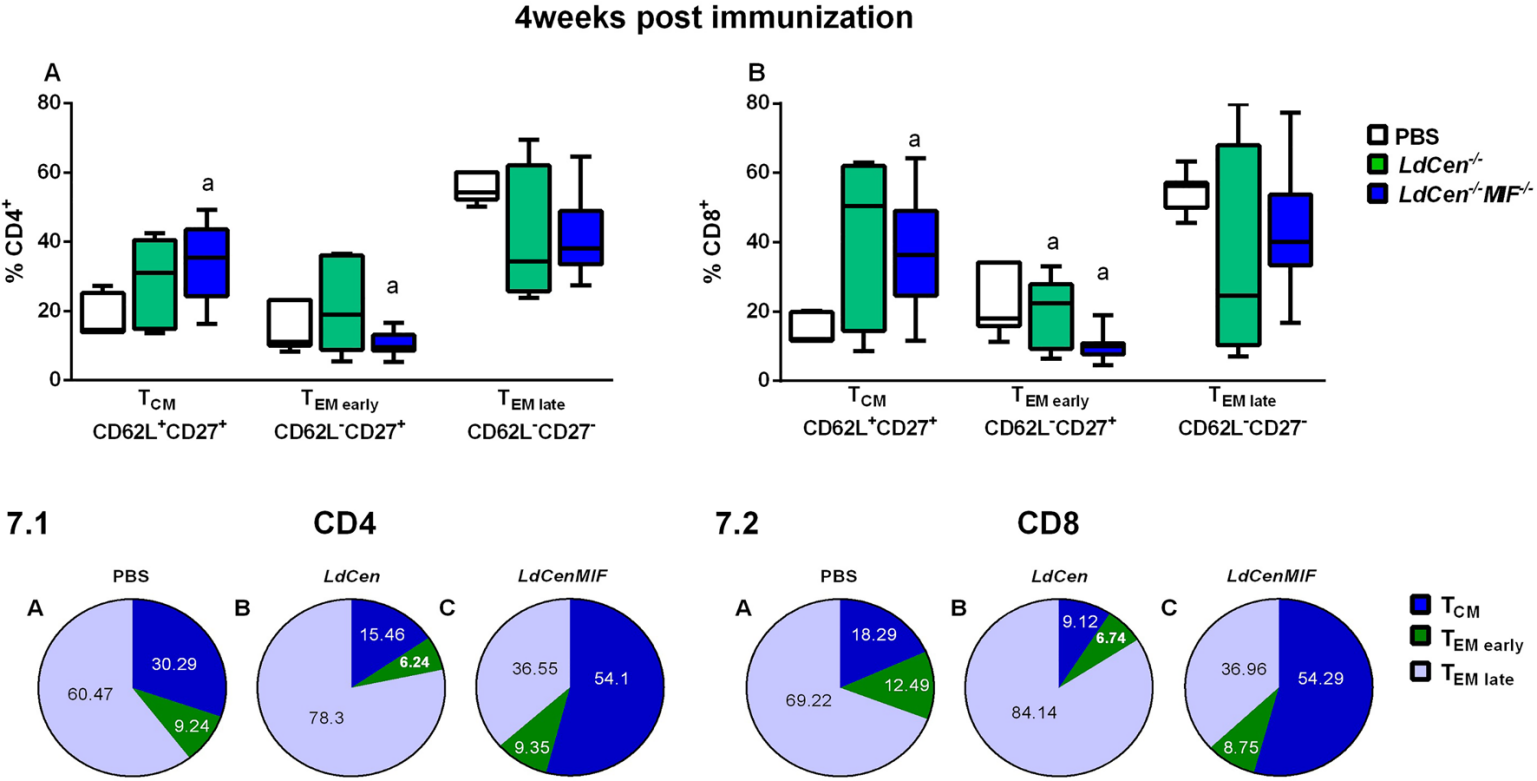
Subtypes of memory T cells 4 weeks post immunization. Expression of CD62L and CD27 molecules by CD3^+^CD4^+^ and CD3^+^CD8^+^ T cells, after specific antigenic stimulation. The profile of the subpopulations of memory cells was assessed as central memory (T_CM_-CD62L^+^CD27^+^), early effector memory (T_EM early_-CD62L^−^CD27^+^) and late effector memory (T_EM late_-CD62L^−^CD27^−^). Results from cultures are expressed as ratio (Percentage of cultures stimulated with SLA *L. infantum*/Percentage of unstimulated cultures-A and B) (Proportion of percentage of cultures stimulated with SLA *L. infantum*/percentage of unstimulated cultures-**7.1** and **7.2**). Significant differences are indicated on the graphs (a: PBS). p values can be found in Supplementary Table 1. The experiments were performed in three replicates and the error bars represent the standard deviation of each group (n = 8 mice/group). The BALB/c mice were evaluated individually.

**Figure 8:**
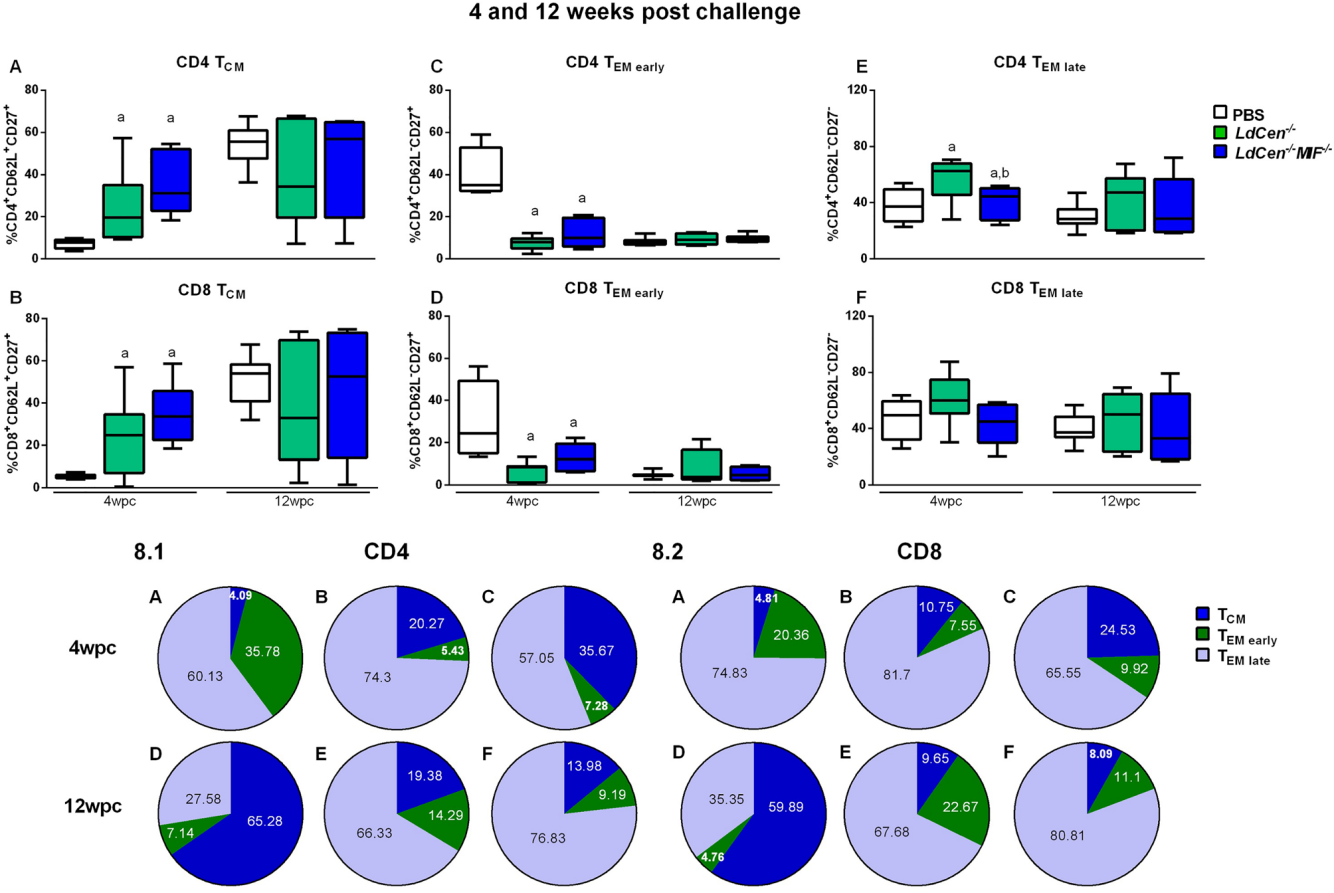
Subtypes of memory T cells 4- and 12-weeks post challenge. Expression of CD62L and CD27 molecules by CD3^+^CD4^+^ and CD3^+^CD8^+^ T cells, after specific antigenic stimulation. The profile of the subpopulations of memory cells was assessed as central memory (T_CM_-CD62L^+^CD27^+^) (A and B), early effector memory (T_EM early_-CD62L^−^CD27^+^) (E and D) and late effector memory (T_EM late_-CD62L^−^CD27^−^) (E and F). Results from cultures are expressed as ratio (Percentage of cultures stimulated with SLA *L. infantum*/Percentage of unstimulated cultures-A and B) (Proportion of percentage of cultures stimulated with SLA *L. infantum*/percentage of unstimulated cultures—**8.1** and **8.2**). Significant differences are indicated on the graphs (a: PBS; b: *LdCen*^*−/−*^). p values can be found in Supplementary Table 2. The experiments were performed in three replicates and the error bars represent the standard deviation of each group (n = 8 mice/group). The BALB/c mice were evaluated individually.

To determine if vaccination with *LdCen*^*−/−*^ or *LdCen*^*−/−*^*MIF*^*−/−*^ attenuated parasites is able to induce memory T cells, we evaluated the expression of CD62L and CD27 molecules by CD3^+^CD4^+^ and CD3^+^CD8^+^ T cells using flow cytometry, after specific antigenic stimulation. The profile of the subpopulations of memory cells was assessed as central memory (T_CM_-CD62L^+^CD27^+^), early effector memory (T_EM early_-CD62L^−^CD27^+^) and late effector memory (T_EM late_-CD62L^−^CD27^−^). The analyses strategy can be observed in supplementary Fig. 3. Analyzing the repertoire of CD4^+^ and CD8^+^ T cells at 4 weeks post immunization, we observed an increased frequency of T_CM_ cells in *LdCen*^*−/−*^*MIF*^*−/−*^ compared to PBS group (p < 0.05) (Fig. 7A,B). After challenge, we demonstrated that immunization with attenuated parasites induced a high frequency of CD4^+^ and CD8^+^ T_CM_ cells in 4wpc compared to PBS group (*LdCen*^*−/−*^: p < 0.05 for both) (*LdCen*^*−/−*^*MIF*^*−/−*^: p < 0001 and 0.01, respectively) (Fig. 8A,B). We observed no statistical differences between groups in 12wpc for CD4^+^ and CD8^+^ T_CM_ cells (Fig. 8A,B).

CD4^+^ and CD8^+^ T_EM early_ showed a decreased frequency of cells in *LdCen*^*−/−*^*MIF*^*−/−*^ group compared to PBS group after 4 weeks post-immunization (p < 0.05 and 0.01, respectively) (Fig. 7A,B). After challenge, we observed that the frequency of CD4^+^ and CD8^+^ T_EM early_ cells significantly decreased in immunized groups compared to PBS group in 4wpc (*LdCen*^*−/−*^: p < 0.01 and 0.05, respectively) (*LdCen*^*−/−*^*MIF*^*−/−*^: p < 0.05 for both), but not in 12wpc (Fig. 8C,D). We also observed that frequency of CD4^+^ T_EM late_ cells decreased in *LdCen*^*−/−*^*MIF*^*−/−*^ group compared to PBS group after 4 weeks post-immunization, but no statistical difference (Fig. 7A), while frequency of CD8^+^ T_EM late_ cells was similar between groups in 4wpi (Fig. 7B). After challenge, immunization with *LdCen*^*−/−*^*MIF*^*−/−*^ followed by 4wpc showed an increase of percentage of CD4^+^ T_EM late_ cells compared to PBS and *LdCen*^*−/−*^ groups (p < 0.05) (Fig. 8E). No differences were observed in frequency of CD8^+^ T_EM late_ cells between groups post challenge (Fig. 8E,F).

We also evaluated the proportion of memory CD4^+^ and CD8^+^ T cells subpopulations 4 weeks post-immunization (Fig. 7.1 and 7.2, Supplementary Table 1) and 4 and 12 weeks after challenge (Figs. 8.1 and 8.2, Supplementary Table 1). The data was represented as parts of whole, to show the fraction of total the subpopulations inside CD3^+^CD4^+^ and CD3^+^CD8^+^ T cells. Mean values of each subpopulation and timepoint is plotted, and the scientific graphic program considers the sum of the populations as 100%. Post-immunization, we observed a similarity between PBS and *LdCen*^*−/−*^ groups, once they demonstrated high proportion of CD4^+^ (Fig. 7.1A,B) and CD8^+^ (Fig. 7.2A,B) T_EM late_ cells in relation to T_CM_ and T_EM early_ cells, respectively. The *LdCen*^*−/−*^*MIF*^*−/−*^ immunized group demonstrated higher fraction of CD4^+^ and CD8^+^ T_CM_ cells compared to T_EM_ cells (early and late) (Fig. 7.1C and 7.2C). After 4 and 12 weeks of challenge, the profile has changed, we observed a predominance proportion of CD4^+^ and CD8^+^ T _EM late_ cells (Fig. 8.1C–F) and CD8^+^ (Fig. 8.2C–F) in *LdCen*^*−/−*^*MIF*^*−/−*^ immunized group.

After 12 weeks of challenge, the groups showed a predominance of distinct memory T cells subpopulations. We showed that CD4^+^ (Fig. 8.1E,F) and CD8^+^ (Fig. 8.2E,F) T_EM late_ cells proportion was higher in both immunized groups compared to PBS group, where CD4^+^ and CD8^+^ T_CM_ cells were majority (Fig. 8.1D and 8.2D).

### Cytokines secretion by memory T cells

**Figure 9:**
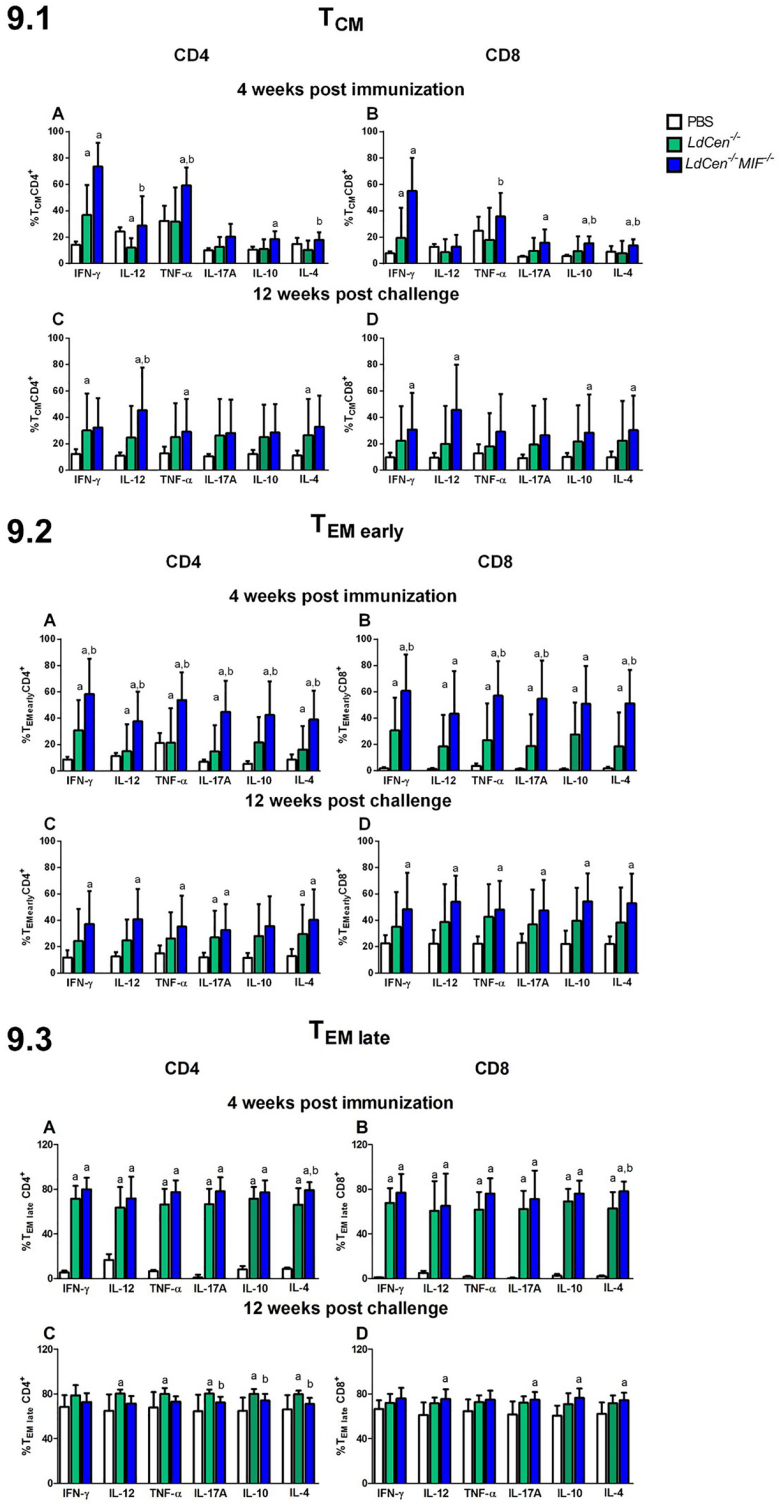
Cytokines production by subtypes of memory T cells. Percentage of IFN-γ, IL-12/IL-23p40, TNF-α, IL-17A, IL-10 and IL-4 production was assessed at 4 weeks post immunization and 12 weeks post challenge in the: (**9.1**) Central Memory T cell subpopulation (T_CM_) of stimulated memory CD4^+^ (A and C) and CD8^+^ (B and D) T cells. (**9.2**) Early Effector Memory T cell subpopulation (T_CM_) of stimulated memory CD4^+^ (A and C) and CD8^+^ (B and D) T cells. (**9.3**) Late Effector Memory T cell subpopulation (T_CM_) of stimulated memory CD4^+^ (A and C) and CD8^+^ (B and D) T cells. Results from cultures are expressed as ratio (percentage of cultures stimulated with SLA *L. infantum*/percentage of unstimulated cultures). Significant differences are indicated on the graphs (a: PBS; b: *LdCen*^*−/−*^). p values can be found in Supplementary Table 3. The experiments were performed in three replicates and the error bars represent the standard deviation of each group (n = 8 mice/group). The BALB/c mice were evaluated individually.

Previous studies have suggested that the balance of pro-and anti- inflammatory cytokines may be associated with protection against leishmaniasis^64–66^**.** IFN-γ, IL-12/IL-23p40, TNF-α, IL-17A, IL-10 and IL-4 production was assessed in the subpopulations of stimulated memory CD4^+^ and CD8^+^ T cells by flow cytometry, in splenocytes isolated from mice immunized with *LdCen*^*−/−*^ and *LdCen*^*−/−*^*MIF*^*−/−*^*,* at 4wpi and 12 wpc (Fig. 9—9.1-T_CM_, 9.2-T_EM early_ and 9.3-T_EM late_). The analyses strategy can be observed in supplementary Fig. 3. p values can be found in supplementary Table 2 (T_CM_), supplementary Table 3 (T_EM Early_), and supplementary Table 4 (T_EM Late_).

In T_CM_ cells (Fig. 9.1), in general, we observed the same profile of cytokine expression in CD4^+^ and CD8^+^ cell compartment. At 4wpi *LdCen*^*−/−*^ and *LdCen*^*−/−*^*MIF*^*−/−*^ groups showed an increase of cytokine production (IFN-γ, TNF-α, IL-10 and IL-4) compared to PBS group (Fig. 9.1A,B). Also, *LdCen*^*−/−*^*MIF*^*−/−*^ immunized mice showed an increase of IL-12/IL-23p40 and TNF-α producing CD4^+^ T cells compared to *LdCen*^*−/−*^ (Fig. 9.1A). *LdCen*^*−/−*^*MIF*^*−/−*^ group presented an increase of CD8^+^ T cells producing most of cytokines but IL-12 (Fig. 9.1B).

Interestingly, at 12wpc the cytokine profile for T_CM_ cells was different. In CD4^+^ T_CM_ cells subpopulation, *LdCen*^*−/−*^*MIF*^*−/−*^ group showed a higher percentage of IL-12p40 expressed by CD4^+^ T cells, compared to *LdCen*^*−/−*^ group (Fig. 9.1C). Meanwhile, CD8^+^ T_CM_ cells showed a higher percentage of IFN-γ, IL-12p40, IL-10 and IL-4, compared to PBS group (Fig. 9.1D), indicating that the manipulated *Leishmania* can address differential activation in different T cell subtypes.

Regarding the expression of cytokines by T_EM early_ (Fig. 9.2), at 4wpi, *LdCen*^*−/−*^*MIF*^*−/−*^ group showed an increase in all evaluated cytokines produced by T cells compared to PBS and *LdCen*^*−/−*^ groups (Fig. 9.2A,B). In CD8^+^ compartment such difference was observed in IFN-g, TNF-a, IL-17A and IL-10 at 4wpi (Fig. 9.2B). Interestingly, at 12wpc significant differences between immunized groups were only seem against PBS group for all cytokines, but IL-10 by CD4^+^ T cells (Fig. 9.2C,D).

Evaluating the T_EM late_ population (Fig. 9.3) at 4wpi, only IL-4 producing cells showed differences between *LdCen*^*−/−*^ and *LdCen*^*−/−*^*MIF*^*−/−*^ groups for both CD4^+^ (Fig. 9.3A) and CD8^+^ (Fig. 9.3B). On the other hand, at 12wpc, *LdCen*^*−/−*^*MIF*^*−/−*^ group showed increased percentages of CD4^+^ (Fig. 9.3C) for all analyzed cytokines, but IFN-γ, compared to *LdCen*^*−/−*^ immunized mice. These phenomena were not kept for CD8^+^ T_EM late_. *LdCen*^*−/−*^*MIF*^*−/−*^ group presented an increase of Il-12p40, IL-17A IL-10 and IL-4, when compared to PBS group (Fig. 9.3D).

### Parasite load

**Figure 10:**
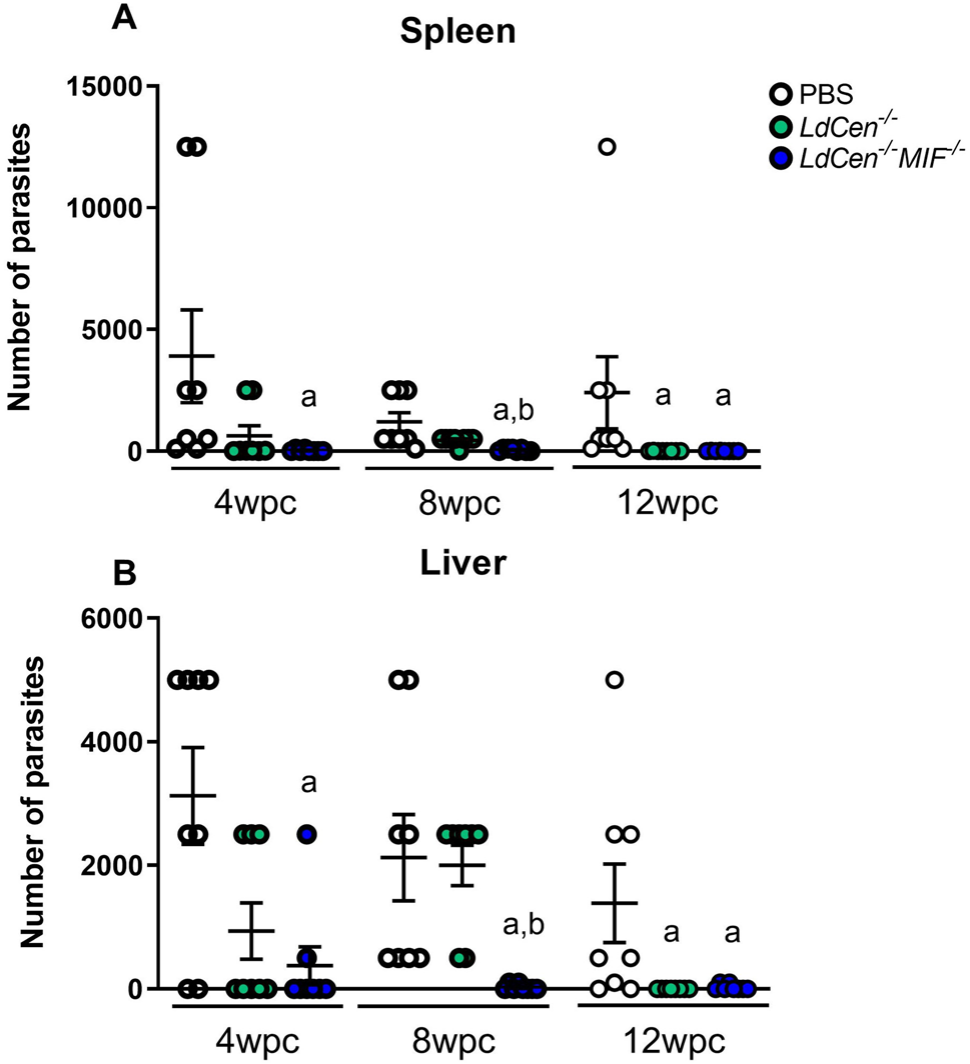
Parasite burden after challenge. Mice (n = 8) were challenged intravenously in the tail with virulent *L*. *infantum* (PP75) and presence of parasites was individually detected 4, 8 and 12 weeks after challenge. The experiments were performed in three replicates and the error bars represent the standard deviation of each group. Spleen and liver from BALB/c mice were used in limiting dilution assay and data expressed as number of parasites/organ. Statistical differences (*p* < 0.05) are indicated in letters (a: PBS; b: *LdCen*^*−/−*^). p values can be found in Supplementary Table 4.

Having observed induction of antibodies production, T cell activation and proliferation, long term memory and cytokines secretion, we evaluated parasitic load in mouse spleen (Fig. 10A) and liver (Fig. 10B) at times 4wpc, 8wpc and 12wpc by serial dilution. At 4wpc, very few *L. infantum* parasites were observed in spleen and liver by *LdCen*^*−/−*^*MIF*^*−/−*^ immunized animals, decreasing the number of parasites observed at 8wpc and 12wpc indicating that the immunization with *LdCen*^*−/−*^*MIF*^*−/−*^ was efficient as a vaccination protocol (Fig. 10). p values can be found in supplementary Table 5.

## Discussion

The development of immunity against *Leishmania* induced by vaccination is widely discussed due to the complexity and antigenic variability of the parasites. Among the models used to study the effect of novel vaccines candidates against *Leishmania* infection, BALB/c and C57BL/6 strains are widely used on those studies^67,68^. The reasons to choose between both strains are: BALB/c mice show an intermediate pattern of activation that favors parasite persistence and chronicity of the disease, while C57BL/6 mice show a classical pattern of activation associated with resolution of the disease^69^. Vaccination using attenuated forms of parasites allow the immune system to interact with a wide repertoire of antigens^69^, inducing a more robust and complete response when compared to recombinant antigen vaccines^70^ because of the presentation of complete array of parasite antigens to the host immune system. In this study, we evaluated a vaccine against visceral leishmaniasis consisting of a double genetically deficient *Leishmania donovani* for the Centrin 1 gene and macrophage migration inhibitory factor (*LdCen*^*−/−*^*MIF*^*−/−*^). The centrin1 gene is associated with cell division, specifically affects the proliferative capacity of amastigote forms that replicate within macrophages but does not affect the replication of promastigote forms. The safety of infection with *LdCen*^*−/−*^ has been previously demonstrated in mice, hamsters and dogs^14–21^. The MIF gene encodes a lymphokine involved in cell-mediated immunity, inhibiting the proliferation of memory cells^71^. It is demonstrated that parasites express MIF-like genes and could interact functionally with the MIF receptor (CD74) acting as evasion mechanism for infection success^59,72–77^. We hypothesized that the use of a parasite knockout for MIF ortholog could induce a long-term memory response, activation and proliferation of B and T cells, inducing cytokines production, and resulting in a cross-protective immunity against *L*. *infantum*. Studies of MIF deletion in *Leishmania major* showed that mice infected with such parasites presented a reduced ability of the parasite to activate antigen-presenting cells, and consequently a reduction in T-cell priming^60^. Also, mice infected with MIF deleted parasites presented a reduction in generation of inflammation and effector CD4^+^ T-cell. Effector CD4^+^ T cells from MIF deleted parasites from infected mice showed a profile of decreased apoptosis, and increased expression of IFN-γ and IL-7R, suggesting that the expression of the orthologue MIF promotes parasite persistence by manipulating the host response to increase the exhaustion and depletion of protective CD4^+^ T cells^60^. However, while low dose infection with wild-type *L. major* parasite is known to result in the acquisition of long-term protection, a process known as leishmanization, after resolution of cutaneous lesions, a persistent infection with *L. major* is established in the host. Thus, this practice has been discontinued due to safety concerns, mainly related to the pathogenicity eventually resulting from uncured lesions, persistence of the parasite in the lesion and reduced vaccine effect (immunosuppression) in patients immunized with vaccine against diphtheria, *Bordetella pertussis* and tetanus^4,5,11^. Based on this concept, Zhang et al.^25^, using a CRISPR genome edited *L. major* strain (*LmCen*^*−/−*^), demonstrated that wildtype *L. major* infected/healed (leishmanization) and *LmCen*^*−/−*^ immunized mice presented high percentage of T CD4^+^ memory cells producing IFN-γ. The low levels of persistent antigens may be important for maintaining long term protection profile, as the generation of IFN-γ producing CD4^+^ T effector populations^25,78^, despite the difference between the survival profile of parasites used in leishmanization and immunization with attenuated parasites. Independently T_CM_ and skin resident T_RM_ memory T cells were also shown to play a role in protection in *L. major* mouse models^39,79^. Thus, it would be hard to discriminate the role of memory cell populations in a *L. major* infection model due to the presence of memory and effector populations and the kinetics of their actions following a challenge infection. Therefore, *LdCen*^*−/−*^*MIF*^*−/−*^ parasites provide an ideal vector to test the role of memory cells in protection considering all characteristics before described here.

The role of the anti-leishmanial antibody response seen in VL is unclear. Some authors have suggested that the presence of anti-leishmanial antibodies could be predictive of disease^80–82^. In the other hand, it has been demonstrated that antibodies against *Leishmania* persist for a long time (> 15 years) after cure and immunity to VL^83^. Moreover, it has a high prevalence of seropositive healthy individuals in areas endemic for VL^84^. Here, we demonstrated that immunization with the double-attenuated strain *LdCen*^*−/−*^*MIF*^*−/−*^ increased the secretion of IgG, IgG_1_ and IgG_2_ (regulated by IL-12 induced production of IFN-γ^85,86^), being able to activate B cells as late as 12 wpc.

The protective immune response against *Leishmania* is mainly mediated by T cells^87^, and the proliferation of these cells is an important indicator of vaccine immunogenicity tested in mice and dogs^88–90^. Our previous work has shown that immunization with *LdCen*^*−/−*^, in dogs and mice, induced T cell proliferation upon stimulation with *Leishmania* antigens^15,16,28^. Consistent with our previous reports, our new study presented here shows that it also happens in mice for *LdCen*^*−/−*^*MIF*^*−/−*^ immunization.

Naïve CD4 T cells post-activation undergo programming for inducible production of cytokines leading to generation of memory cells with various functions. The importance of the Th1/Th2 balance in the outcome of leishmaniasis has been demonstrated by many studies^91–95^. Peine and collaborators^96^ described that hybrid Th1/2 cells arise naturally during parasite infections and that the two opposing differentiation programs can stably co-exist in resting memory for months, demonstrating a cell-intrinsic self-limiting mechanism that can prevent excessive inflammation. These facts corroborate our findings with an increased mixed cytokines production by T cells after immunization and a persistent of IL-12 production by double-mutant parasites after challenge leaded to a protective profile seem in this study.

One of the most crucial aspects of vaccination is the understanding of the T cell memory profile required to obtain effective vaccines against parasites and virus. T cell memory is the ability of a population to respond to a challenge, recognizing an antigen by its receptors (TcRs). T cell response occurs after the initial exposure to the antigen, by proliferating and/or expressing molecules capable of mediating an effector reaction. A pertinent memory is associated with protection against infection and/or disease when challenged with a pathogen in experimental model^97^. Briefly, naïve T cells (TN) respond to antigenic peptides complexed to major histocompatibility complex (MHC) molecules on antigen-presenting dendritic cells (DCs) after an initial antigenic exposure and priming. After priming, part of those cells proliferates and become effector-memory T cells (T_EM_), losing the molecules and CD45RA in the process, and being held back in secondary lymphoid tissues (SLT)^98^. Prompt protection is conferred by tissue resident or circulating effector memory T cells (T_EM_) that survey frontline barriers and affected tissues for incoming pathogens and exhibit immediate effector role upon antigen recognition. Another portion of primed cells become central memory T cells (T_CM_), responsible for recalling responses and to patrol the T cell areas of secondary lymphoid tissues, where they can quickly proliferate in response to antigens^98^. Those circulating quiscent cells are a provision that can respond to the re-encounter with an antigen within SLT by proliferating and differentiating into T_EM_ and T_Eff_ cells over the course of few days. Central memory T cells (T_CM_) are considered better applicable to protect against pathogens with longer incubation periods, such as protozoans^97^. Studies testing single or polyproteins recombinant proteins from *Leishmania* showed that immunization successfully generate antigen-specific cells that exhibit characteristics of T_CM_, cytokine production upon antigen re-exposure and increased Th1 response upon challenge compared to nonimmunized animals^43,99–102^. In addition, it has been demonstrated that parasites can use MIF ortholog to actively modulate the host immune response, preventing the development of effective memory CD4^+^ T cells^61^. Our data demonstrated that immunization with double-attenuated parasites induced T_CM_ cells, while PBS control and *LdCen*^*−/−*^ groups presented high percentage of T_EM_ cells after immunization. Interestingly, the percentage of T_CM_ cells of *LdCen*^*−/−*^*MIF*^*−/−*^ group after challenge decreased while T_EM Late_ cells increased. It suggests a conversion of the memory subtype using the double-deletion mutant parasites. Thus, the deletion of MIF gene in *LdCen*^*−/−*^ attenuated parasites could yield a long-lasting immune response, suggested by the increase of T_CM_ cells after immunization.

The evaluation of parasite load allows us to visualize not only number of parasites, but also viability of the parasite. The level of parasite burden in spleen and liver observed in both *LdCen*^*−/−*^ and *LdCen*^*−/−*^*MIF*^*−/−*^ immunized groups is decreased compared to the positive control (animals immunized with PBS and challenged) group at 12wpc, suggesting a robust degree of protection. Therefore, the protection obtained in the present study confirms the ability of *LdCen*^*−/−*^*MIF*^*−/−*^ and *LdCen*^*−/−*^ vaccines to limit parasite replication and prevent severe disease after challenge. Okwor and colleagues^103^, evaluating the differences in the immune responses to live and killed *L. major* in experimental model, have demonstrated that both are qualitatively different. The data demonstrated that live attenuated parasite induced strong and durable protection against virulent secondary challenges^103^, indicating that is a good way to achieve protective immunity against *Leishmania* infection by vaccination. In our previous work, vaccination with the attenuated parasite proved to be safe, protective and persistent in mice (BALB/c and SCID), hamsters and dogs, after challenge with wild forms, in addition to cross-protection in animals challenged with *L. braziliensis*, *L. infantum*^14–18,27,40^. In addition, *LdCen*^*−/−*^*MIF*^*−/−*^ immunized mice appear to clear infection in spleen and liver sooner than those immunized with *LdCen*^*−/−*^ parasites.

Overall, the results indicate that the combination of deletion of Centrin and MIF genes produced a prominent immunological response, inducing central memory T cells (long-term immune response) after immunization, T and B cell activation, balanced cytokine production and protection against challenge with wild type strain. Despite the deletion only for MIF gene does not seem to affect growth or replication of the parasite, it does seem to affect the virulence factor. These finding points to the fact that the induction of the profile of memory cells are necessary for a protective response and provide novel insights into developing vaccines against pathogens. The results indicate that *LdCen*^*−/−*^*MIF*^*−/−*^ attenuated parasites are potential candidates for the development of an attenuated vaccine against leishmaniasis.

## Methods

### Expression of MIF proteins in *E. coli*

*Leishmania donovani* MIF1 and MIF2 ORFs were amplified by PCR and ligated into pCR T7/CT-Topo (Invitrogen). The recombinant proteins were expressed from *E. coli* and purified in native conditions through Ni-agarose column chromatography.

### MIF induced TNF-α and IL-12 production by BMDCs

BMDCs were incubated in presence of purified recombinant LdMIF1 and LdMIF2 proteins (10 ng/ml) and LPS (0.25 µg/mL) for 24 h and the culture supernatants were used for measuring TNF-α by ELISA. To test the production of IL-12 by BMDCs in presence of recombinant LdMIF1 protein, cells were incubated in presence of an increasing concentrations of rLdMIF1 (0–100 ng/ml) for 24 h and IL-12 production in the culture supernatants was measured by ELISA. The experiment was performed four times (n = 3/replication), and cells were pooled.

### Deletion of MIF genes

*Leishmania infantum* genome contains two homologs of MIF gene on chromosome 33 (LINF_330025900 and LINF_330026000) each encoding a 342 bp ORF. The drug resistance markers Blasticidin and Puromycin were used to obtain *LdCen*^*−/−*^*MIF*^*−/−*^. The PvuII restriction site in the Blasticidin ORF was altered by PCR prior to incorporating in the targeting construct. To generate the targeting construct, a 462 bp fragment from the 5′ region and an 834 bp fragment from the 3′ region flanking the *L. infantum* MIF open reading frames were amplified by PCR using *L. infantum* genomic DNA. The primers used to amplify 5′ flanking fragment included restriction sites HindIII and BamHI. Similarly, the primers added SpeI and XbaI sites to the 3′ flanking fragment. The drug resistance markers blasticidin (BSD) and puromycin (PAC) were amplified with primers that add BamHI and SpeI to the open reading frame. These DNA fragments were subcloned into the pCR2.1-Topo vector and the nucleotide sequence was determined to ensure fidelity. The plasmid containing the 5′ flanking fragment was digested with HindIII/BamHI, gel purified and ligated into a similarly digested plasmid containing either blasticidin or puromycin. The resultant plasmids, containing both the 5’flanking region and the drug resistance markers were digested with SpeI/XbaI and the 3’flanking fragment isolated by SpeI/XbaI digestion was ligated into these sites. The authenticity of the final plasmid was confirmed by DNA sequencing. For the purpose of transfection, the targeting construct was prepared by digestion with HindIII/XbaI, which releases a linear fragment containing the MIF 5′ flanking sequence, the blasticidin/puromycin encoding DNA fragments and the MIF 3′ flanking sequence. The fragment was gel purified and used in transfection. SalI digested genomic DNA from *LdWT*, *LdCen*^*−/−*^, *LdCen*^*−/−*^*MIF*^*−/−*^ parasite clones selected on Nobel agar plates was resolved on agarose gels. The blots from these gels were probed with ^32^P labeled probes corresponding to Centrin, Neomycin, Hygromycin, MIF, Puromycin and Blasticidin ORFs. The 168 bp MIF probe selected corresponds to c’end of the ORF and is common to both MIF1 and MIF2 genes. The visualization was made by autoradiograph. Figures 2B, 4A,B were visualized in different days, but visualized by autoradiograph with no exposure differences.

### IFN-γ expression

Balb/C mice were intravenously infected with 3 × 10^6^ stationary phase *LdWT* or *LdMIF*^*−/−*^ parasites and spleens were collected on 5- and 7-days post-infection. The cells were stimulated with 0.25 µg/mL of lipopolysaccharide (LPS). Expression of IFN-**γ** was measured by ELISA from the culture supernatants. The experiment was performed four times (n = 3/replication), and cells were pooled.

### Macrophage infection

C57BL/6 murine bone marrow derived macrophages were cultured in RPMI medium containing 10% FBS macrophage colony-stimulating factor (20 ng/ml, ProSpec, Israel), plated in 0.5 ml on eight-chamber Lab-Tek tissue-culture slides (Miles Laboratories). The differentiated macrophages were infected with *LdWT*, *LdCen*^*−/−*^, *LdMIF*^*−/−*^ and *LdCen*^*−/−*^*MIF*^*−/−*^ stationary phase promastigote cultures (10:1 parasite-to-macrophage ratio). Free extra cellular parasites were aspirated after 6 h incubation at 37 °C in 5% CO2 and the cultures were incubated in macrophage culture medium for 7 days. The parasite counts were measured by using Diff-Quick Stain (Baxter Healthcare Corporation). A minimum of 300 macrophages were counted. The results are expressed as the number of amastigotes per 100 macrophages. The experiment was performed four times (n = 3/replication), and cells were pooled.

### T cell apoptosis

Balb/C mice were infected intravenously with 3 × 10^6^ stationary phase *LdWT, LdMIF*^*−/−*^ or *LdCen*^*−/−*^*MIF*^*−/−*^ parasites. Spleens were collected on 5-, 9-, and 14-days post-infection. Splenocytes were stained with 7AAD, CD3-Alexaflour700, CD4-Pacific blue, CD8-BV650 and Annexin-V-PE antibodies and analyzed on BD LSR-Fortessa.

### Parasites and soluble antigen (SLA) preparation

The *L. donovani* centrin1-deleted (*LdCen*^*−/−*^) and centrin1 and MIF-deleted (*LdCen*^*−/−*^*MIF*^*−/−*^) parasites were used for immunization and maintained as previously described^23^. *L. infantum* promastigote forms (MHOM/BR/1972/PP75) were grown as described^104^. For preparation of SLA, *L. infantum* stationary-phase promastigotes were harvested, washed three times in PBS and ruptured using a cell disruptor (Sonifier Cell Disruptor, Branson Sonic Power Co., Danbury, CT, USA). The ruptured parasite suspension was centrifuged at 18,500 rpm for 90 min at 4 °C. The supernatant was dialyzed against PBS for 24 h and sterilized by filtration through 0.22 µm syringe filters and stored at − 80 °C. Protein quantification was performed using Pierce^®^ BCA Protein Assay Kit (Thermo Scientific, USA) as described by the manufacturer.

### Animals and vaccination protocol

Animal studies were carried out in accordance with the recommendations in the Guide for the Care and Use of Laboratory Animals of the National Institutes of Health and the guidelines set by the Brazilian Animal Experimental College (COBEA). For studies in Brazil, the protocol was reviewed and approved by the Ethical Committee for the Use of Experimental Animals of the Oswaldo Cruz Foundation (CEUA/FIOCRUZ Protocol LW35/14). The animal protocol for the studies in USA has been approved by the Institutional Animal Care and Use Committee at the Center for Biologics Evaluation and Research, US Food and Drug Administration (FDA) (ASP 1995#26). In addition, the animal protocol is in full accordance with “The guide for the care and use of animals as described in the US Public Health Service policy on Humane Care and Use of Laboratory Animals 2015.” This study is reported in accordance with ARRIVE guidelines. All animals were certified free of endo/ectoparasites by animal house logistics. Female 6- to 8-week-old C57Bl/6 (Jackson labs) mice were immunized or infected, intravenously with 3 × 10^6^ total stationary phase promastigotes of *LdMIF*^*−/−*^ or LdWT parasites in 10 μl PBS. The mice, with 5–7 weeks of age were divided into three groups (8 animals per group/time). *LdCen*^*−/−*^ and *LdCen*^*−/−*^*MIF*^*−/−*^ groups received intravenously 3 × 10^6^ *LdCen*^*−/−*^ or *LdCen*^*−/−*^*MIF*^*−/−*^ promastigotes at stationary phase, respectively. Control group received PBS alone. Four weeks after immunization, all animals (including PBS group) were challenged with 3 × 10^6^ *L. infantum* parasites. The immunological parameters were measured 4 weeks post-immunization (4wpi), 4, 8 and 12 weeks (wpc) after the challenge with 3 × 10^6^ of stationary phase promastigotes of *L. infantum* intravenously, as demonstrated in Supplementary Fig. 1. Regarding antibody response, T cell proliferation, intracellular cytokine measurement, subtype of memory and parasite load experiments, we performed the experiments three times. The BALB/c mice (n = 8/group/time point, three replicates). were individually assessed, using the same animals for all those experiments mentioned before.

### Antibody responses

Antigen-specific IgG_Total_, IgG_1_ and IgG_2_ levels were measured by indirect ELISA^64^. Briefly, 96 wells micro titer plates (Nalgen Intl., USA) were coated overnight with 5 µg/mL of SLA. For IgG_Total_, IgG_1_ and IgG_2_ analysis, sera were added at a 1:100 dilution. Peroxidase-conjugated rabbit anti-mouse IgG_Total_ (1:3000), IgG_1_ (1:2000) or IgG_2_ (1:1000) antibodies were added for 1 h. The reaction was developed using TMB (Sigma, USA) and H_2_SO_4_ stop solution was used. Absorbance was measured on VersaMax 340PC microplate reader (Molecular Devices, USA) at 450 nm.

### Flow cytometric analysis of phenotypic profile and intracytoplasmic cytokine production

Spleens were sterilely removed, and single cell suspensions prepared. Mononuclear cells were enumerated using a Countess Automated Cell Counter (Thermo Fischer, Invitrogen, MA). Cells were cultured at 2 × 10^5^ cells per well in duplicate in a 96 wells plate (Corning Incorporated, Corning, NY) in RPMI-1640 supplemented with 10% heat-inactivated FBS, 50,000 Units penicillin/streptomycin (Invitrogen) and 1% l-glutamine (Gibco). The cells were incubated in the presence or absence of 25 µg/mL SLA at 37 °C in 5% CO_2_ for 72 h. On the last 4 h, cultures received brefeldin A (10 µg/mL). Cells were stained for the surface markers, all purchased from Biolegend (CA,USA): (a) CD3 FITC (clone 145–2C11), (b) CD3 AlexaFluor700 (clone 17A2), (c) CD4 PerCP-Cy5.5 (clone RM4-4), (d) CD8 BV421 (clone 53–6.7), (e) CD62L BV605 (clone MEL-14), (f) CD27 PE-Cy7 (clone LG.3A10), (g) CD25 BV510 (clone PC61), (h) GATA3 AlexaFluor647 (clone 16E10A23), (i) T-bet PE-Cy7 (clone 4B10). Cells were then fixed, permeabilized and stained for the following cytokines: (a) IL-4 (clone 11B11), (b) IL-12p40 (clone C15.6), (c) IFN-γ (clone XMG1.2), (d) TNF-α (clone MP6-XT22), (e) IL-5 (clone TRFK5), (f) IL-17A (clone TC11-18H10.1) and (g) IL-10 (clone JES5–16E3), all PE. For each sample, at least 100,000 cells were analyzed. The data were analyzed using FlowJo software and a FACSFortessa flow cytometer (both from Becton Dickinson, San Jose, CA).

### In vitro proliferative response of lymphocytes

Splenocytes were isolated as described above. After 72 h incubation, cell proliferation analysis was performed on splenocytes labeled with Ki-67 APC (clone16A8, Biolegend, CA) essentially as described above, and analyzed the stimulation index using monoclonal antibodies CD3, CD4 and CD8. In this sense, proliferation responses were expressed in terms of stimulation ratio that was calculated as: mean proliferation response of cultures stimulated SLA *L. infantum*/mean proliferation response of unstimulated cultures as described previously^15^.

### Determination of parasite burden

At 4 weeks post immunization, and 4-, 8- and 12-weeks post challenge, the parasite load was measured in the spleen and liver by the limiting dilution assay as previously described^105^.

### Statistical analysis

Statistical analysis was performed using GraphPad Prism 6.0 software (GraphPad Software Inc, USA). Non-parametric Kruskal–Wallis test followed by Dunns test was used to compare data from all three groups (*LdCen*^*−/−*^, *LdCen*^*−/−*^*MIF*^*−/−*^ and PBS). Differences were considered significant when a *p* value ≤ 0.05 was obtained.

## Supplementary Information

Supplementary Information.

## Data Availability

The datasets used and/or analyzed during the current study and supporting the conclusions of this article are included in this article. These datasets are also available from the corresponding author on reasonable request.
