# Supplementary material for: Deletion of MIF gene from live attenuated LdCen−/− parasites enhances protective CD4+ T cell immunity
Source: Sci Rep. 2023 May 5;13:7362. doi: 10.1038/s41598-023-34333-2 (PMC10163264; doi:10.1038/s41598-023-34333-2)
Supplement: Supplementary file 1 — Supplementary Information. [file 41598_2023_34333_MOESM1_ESM.docx]

**
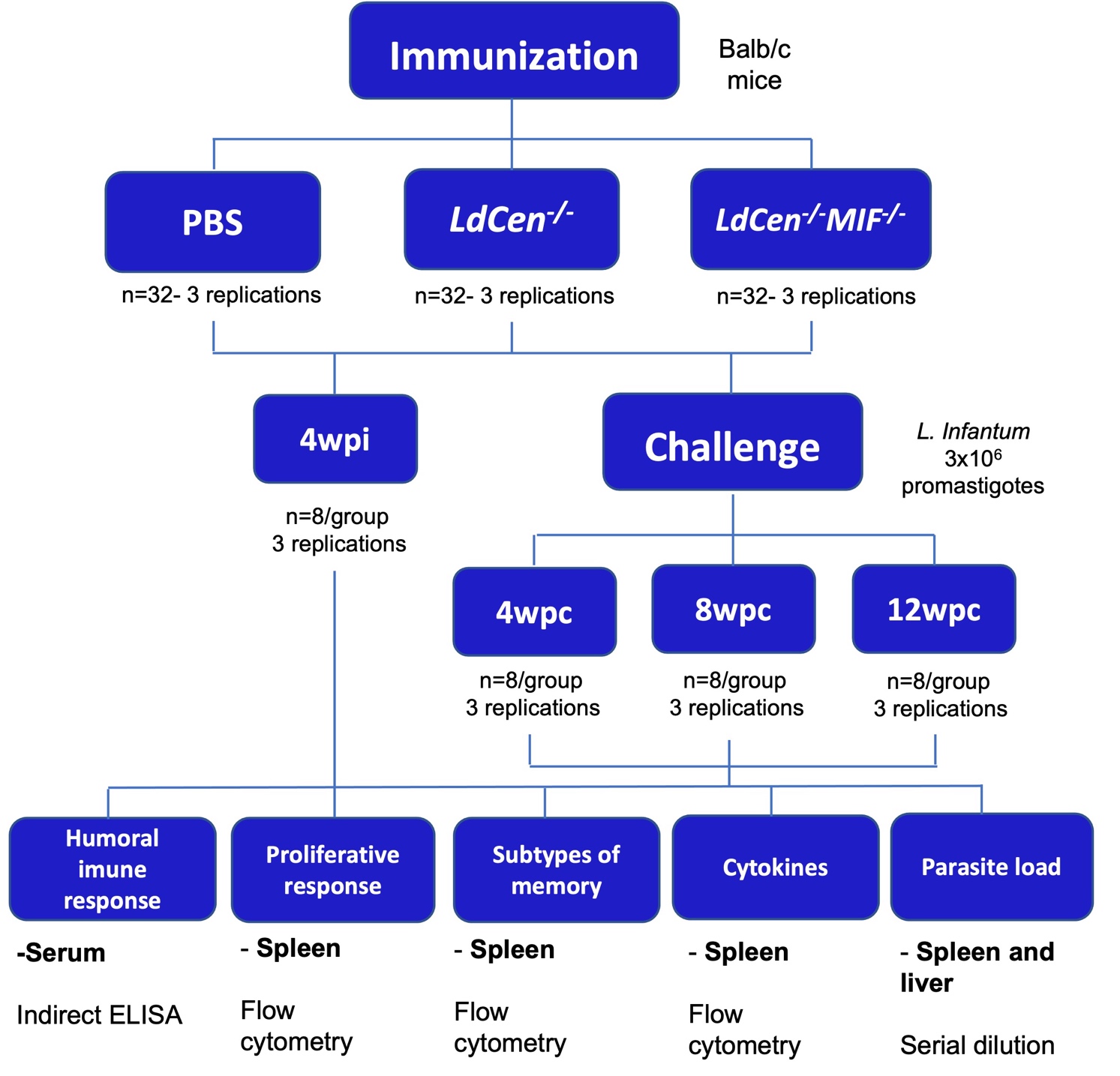
Supplementary Figure 1. Schematic design of antibody response, T cell proliferation, intracellular cytokine measurement, subtype of memory and parasite load experiments.** The experiments were performed three times. The BALB/c mice (n=8/group/time point) were individually assessed, using the same animals for all those experiments. wpi-weeks post-immunization; wpc-weeks post-challenge.

**
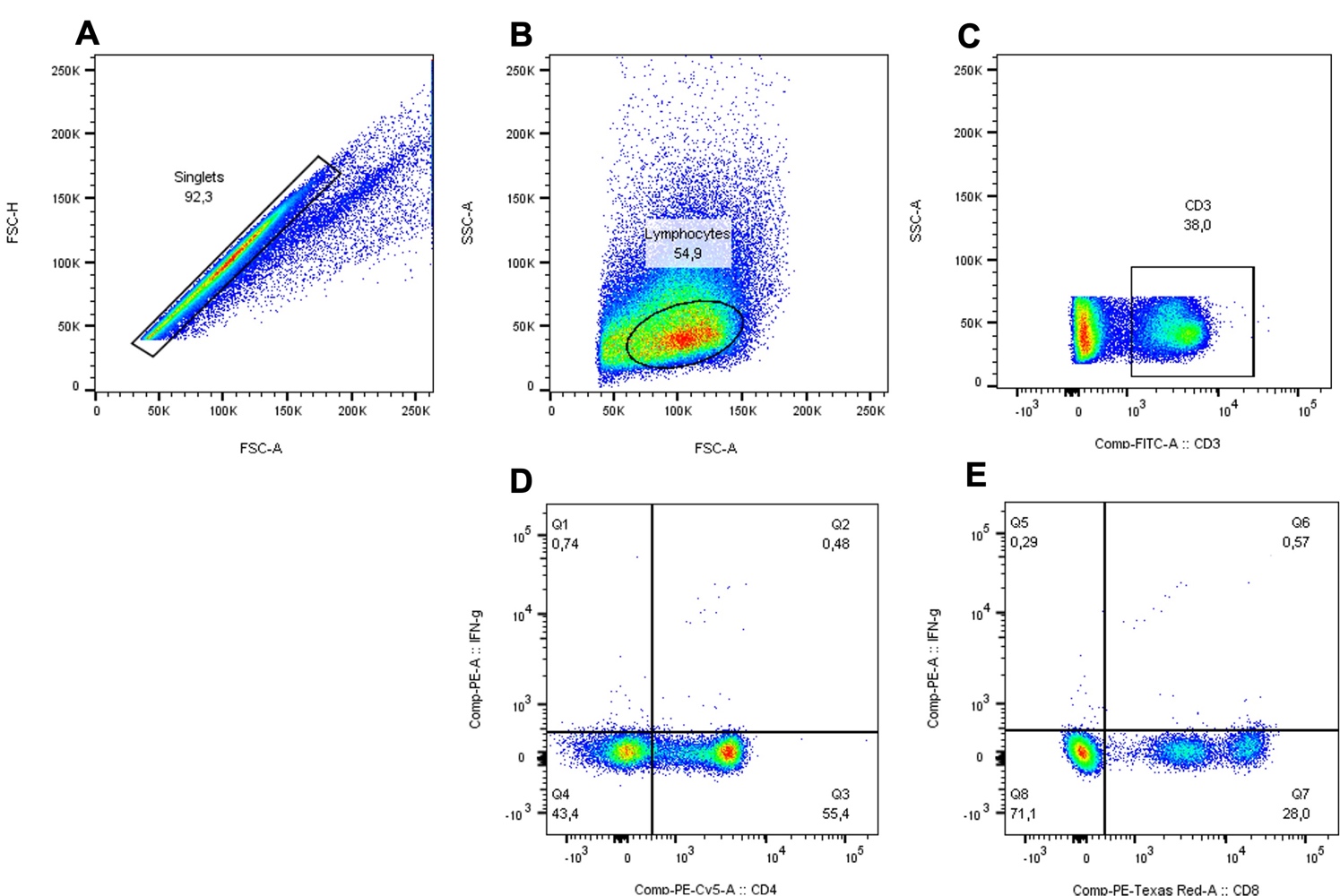
**

**Supplementary Figure 2. Analysis Strategy for Intracellular cytokines expression by T cells.** (A) Size gate FSC-H x FSC-A to select single cells (B) Identification of peripheral lymphocytes population in diagram of FSC×SSC. (C) Dot plot of SSC-A×CD3 to select T cells. (D) Dot plot of CD4×IFN-γ to select CD4^+^ T cells producing cytokines and (E) Dot plot of CD8×IFN-γ to select CD8^+^ T cells producing cytokines.

**
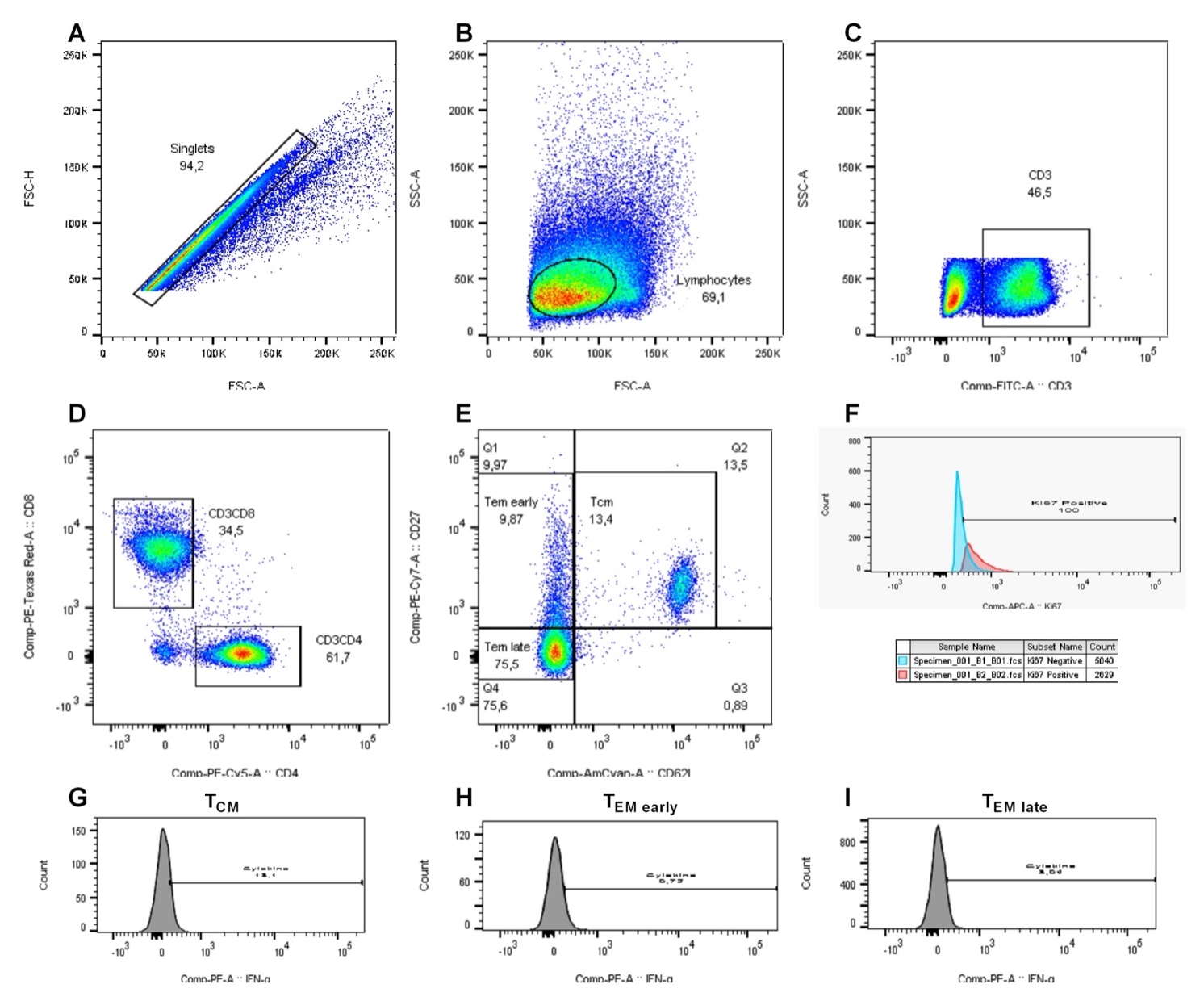
**

**Supplementary Figure 3. Analysis Strategy for Intracellular cytokines expression by Subtypes of Memory T cells and proliferation.** (A) Size gate FSC-H x FSC-A to select single cells (B) Identification of peripheral lymphocytes population in diagram of FSC×SSC. (C) Dot plot of SSC-A×CD3 to select T cells. (D) Dot plot of CD4×CD8 to select CD4^+^ and CD8^+^ T cells. (E) Dot plot of CD62L×CD27 displaying the frequency of subpopulations of memory cells, assessed as central memory (T_CM_-CD62L^+^CD27^+^), early effector memory (T_EM early_-CD62L^-^CD27^+^) and late effector memory (T_EM late_-CD62L^-^CD27^-^). (E) Medium Intensity of Fluorescence (MIF) of Ki67 to evaluate T cell proliferation. (G, H and I) Histrogram of IFN-γ displaying the frequency of cytokines produced by T_CM_, T_EM Early_ and T_EM Late_.

**Supplementary table 1- Proportion of Central Memory T cells (T_CM_), Early Effector Memory T cells (T_EM early_) and Late Effector Memory T cells (T_EM late_), represented as percentage values**

| **Time point** | **T cell** | **Group** | **TCM (%)** | **TEM early (%)** | **TEM late (%)** |
| --- | --- | --- | --- | --- | --- |
| 4wpi | CD4 | PBS | 30.29 | 9.24 | 60.47 |
|  |  | *LdCen-/-* | 15.46 | 6.24 | 78.3 |
|  |  | *LdCen-/-MIF-/-* | 54.1 | 9.35 | 36.55 |
|  |  |  |  |  |  |
|  | CD8 | PBS | 18.29 | 12.49 | 69.22 |
|  |  | *LdCen-/-* | 9.12 | 6.74 | 84.14 |
|  |  | *LdCen-/-MIF-/-* | 54.29 | 8.75 | 36.96 |
|  |  |  |  |  |  |
| 4wpc | CD4 | PBS | 4.09 | 35.78 | 60.13 |
|  |  | *LdCen-/-* | 20.27 | 5.43 | 74.3 |
|  |  | *LdCen-/-MIF-/-* | 35.67 | 7.28 | 57.05 |
|  |  |  |  |  |  |
|  | CD8 | PBS | 4.81 | 20.36 | 74.83 |
|  |  | *LdCen-/-* | 10.75 | 7.55 | 81.7 |
|  |  | *LdCen-/-MIF-/-* | 24.53 | 9.92 | 65.55 |
|  |  |  |  |  |  |
| 12wpc | CD4 | PBS | 65.28 | 7.14 | 27.58 |
|  |  | *LdCen-/-* | 19.38 | 14.29 | 66.33 |
|  |  | *LdCen-/-MIF-/-* | 13.98 | 9.19 | 76.83 |
|  |  |  |  |  |  |
|  | CD8 | PBS | 59.89 | 4.76 | 35.35 |
|  |  | *LdCen-/-* | 9.65 | 22.67 | 67.68 |
|  |  | *LdCen-/-MIF-/-* | 8.09 | 11.1 | 80.81 |
|  |  |  |  |  |  |

wpi: weeks post immunization; wpc: weeks post challenge

**Supplementary table 2- p values of cytokines secretion by Central Memory T cells (T_CM_)**


wpi: weeks post immunization; wpc: weeks post challenge

**Supplementary table 3- p values of cytokines secretion by Early Effector Memory T cells (T_EM early_)**

wpi: weeks post immunization; wpc: weeks post challenge

**Supplementary table 4- p values of cytokines secretion by Late Effector Memory T cells (T_EM late_)**

wpi: weeks post immunization; wpc: weeks post challenge

**Supplementary Table 5- p values of parasite load in spleen and liver**

|  |  | **Parasite load** | |  |
| --- | --- | --- | --- | --- |
| **Time** | **Spleen** | **p value** | **Liver** | **p value** |
| 4wpc | PBS x *LdCen^-/-^MIF^-/-^* | 0.0015 | PBS x *LdCen^-/-^MIF^-/-^* | 0.032 |
|  |  |  |  |  |
| 8wpc | PBS x *LdCen^-/-^MIF^-/-^* | 0.0006 | PBS x *LdCen^-/-^MIF^-/-^* | 0.0017 |
|  | *LdCen^-/^*^-^ x *LdCen^-/-^MIF^-/-^* | 0.0148 | *LdCen^-/^*^-^ x *LdCen^-/-^MIF^-/-^* | 0.001 |
|  |  |  |  |  |
| 12wpc | *PBS x LdCen^-/^*^-^ | 0.0003 | *PBS x LdCen^-/^*^-^ | 0.0035 |
|  | PBS x *LdCen^-/-^MIF^-/-^* | 0.0011 | PBS x *LdCen^-/-^MIF^-/-^* | 0.0431 |
|  |  |  |  |  |
